# Non-Invasive Prenatal Screening for Down Syndrome: A Review of Mass-Spectrometry-Based Approaches

**DOI:** 10.3390/life15050695

**Published:** 2025-04-24

**Authors:** Răzvan Lucian Jurca, Ioana-Ecaterina Pralea, Maria Iacobescu, Iulia Rus, Cristina-Adela Iuga, Florin Stamatian

**Affiliations:** 1 Mother and Child Department, Obstetrics and Gynecology I, “Iuliu Hațieganu” University of Medicine and Pharmacy Cluj-Napoca, 400347 Cluj-Napoca, Romania; jurca.razvan.lucian@elearn.umfcluj.ro; 2 Personalized Medicine and Rare Diseases Department, MEDFUTURE—Institute for Biomedical Research, “Iuliu Hațieganu” University of Medicine and Pharmacy Cluj-Napoca, Louis Pasteur Street 6, 400349 Cluj-Napoca, Romaniamaria.iacobescu@medfuture.ro (M.I.); 3 Department of Clinical Pharmacy, Faculty of Pharmacy, “Iuliu Hațieganu” University of Medicine and Pharmacy Cluj-Napoca, 400349 Cluj-Napoca, Romania; rus.iulia@umfcluj.ro; 4 Department of Pharmaceutical Analysis, Faculty of Pharmacy, “Iuliu Hațieganu” University of Medicine and Pharmacy Cluj-Napoca, 400349 Cluj-Napoca, Romania; 5 Imogen Clinical Research Centre, 400347 Cluj-Napoca, Romania

**Keywords:** Trisomy 21, Down syndrome, prenatal screening, mass-spectrometry-based omics, proteomics, metabolomics

## Abstract

Down Syndrome or Trisomy 21 (T21) is a complex genetic disease characterized by the presence of an extra chromosome 21, which leads to multiple clinical features and manifestations that severely affect the patient’s quality of life. Various methods of prenatal screening have been developed over time, allowing informed decision-making. However, a common drawback of the current methods for detecting T21 is their invasive nature. Over the past years, mass-spectrometry-based omics technologies have become a key tool for discovering biomarkers for the prenatal screening of T21, particularly focusing on proteins, peptide sequences, or metabolites in samples, like amniotic fluid, umbilical cord blood, and others. Recently, there has been a noticeable shift towards using less invasive biological sample types (e.g., maternal serum, plasma, and urine) reflecting a growing interest in non-invasive methods for prenatal screening. These advances aim to improve the sensitivity and accuracy for T21 detection while reducing the risks associated with more invasive procedures. The first section of this paper offers an in-depth review of studies utilizing mass-spectrometry-based omics for the prenatal screening of T21. This part provides an overview of the methodologies employed and their key findings. Instead, the subsequent section offers a comprehensive examination of the differentially expressed proteins (DEPs) and metabolites (DEMs) reported in the literature in T21 prenatal screening. Additionally, pathway analysis is carried out to explore the biological pathways that these molecules are involved in and how they relate to the clinical features of the syndrome. These findings aim to guide future research in the field and foster the development of more advanced, less invasive prenatal screening techniques for T21.

## 1. Introduction

Down syndrome, also referred to as Trisomy 21 (T21), is the most prevalent chromosomal abnormality, with an incidence of about 10.1 in 10,000 live births in Europe based on the estimated number between 2011 and 2015 [1]. In the United States, the reported occurrence is about 1 in every 640 births [2]. T21 is caused by the presence of an extra copy of chromosome 21 in some or all cells of an individual. The genetic anomaly leads to distinctive clinical features, including cognitive and developmental delays, physical abnormalities, and an increased susceptibility to specific medical conditions [3,4].

Advancements in prenatal screening for T21 have significantly improved over the years, driven by the development of diverse screening methods. The initial approach, implemented in the second trimester, was the Triple Test, which measures maternal serum levels of alpha-fetoprotein (AFP), total human chorionic gonadotropin (hCG), and unconjugated estriol (uE3), alongside maternal age as a risk factor. Later, the Quadruple Test added inhibin-A as a second-trimester marker, enhancing the detection accuracy [5,6]. In the 1990s, the first trimester combined test was introduced, incorporating ultrasound markers, such as nuchal translucency thickness, and biochemical markers, like pregnancy-associated plasma protein A (PAPP-A) and free β-hCG, allowing for earlier detection between 10 and 13 weeks of gestation [5,6]. The integrated test, which combines markers from both trimesters, subsequently provided even more refined screening [5,6]. Despite these advancements, earlier methods had limitations, particularly in accuracy, underscoring the need for continuous improvements. Several reviews concerning prenatal screening for T21 have been published over time, with some addressing the etiopathophysiology and complications associated with the condition [3], while others focused exclusively on the available methods for prenatal screening [7,8,9]. Furthermore, there has been an analysis of the rates of pregnancy termination after a positive result from prenatal screening [10]. The topic of parental education regarding prenatal screening has also been explored, proposing different ways to provide information that aids prospective parents in making informed decisions [11]. A more recent review has emphasized the role of omics sciences in the prenatal diagnosis of T21, particularly highlighting advancements in genomics approaches [12]. The early detection of T21 during pregnancy is vital for enabling informed decision-making by expectant parents. Knowing the T21 status early allows families to (1) access genetic counseling, which offers information about T21 and support in exploring options, including planning for the baby’s arrival if the pregnancy is continued; (2) develop a birth plan addressing potential medical concerns; and (3) opt for safer procedures if the termination of pregnancy is chosen, typically before fetal viability [13]. Current prenatal screening methods for T21 provide valuable risk assessments but do not serve as conclusive diagnostic tools. Approaches, such as maternal serum screening and first-trimester combined tests, can indicate an increased risk of T21 [14]. A conclusive diagnosis generally requires invasive procedures, such as amniocentesis and chorionic villus sampling (CVS). These methods involve extracting fetal cells by puncturing the amniotic sac (amniocentesis) or the placenta (CVS) to conduct a chromosomal analysis. While highly accurate, these invasive methods carry a small risk of miscarriage, typically less than 1% [15]. This risk, combined with the psychological stress associated with the procedures, represents a significant limitation for some pregnant women. The development of non-invasive prenatal testing (NIPT) using the cell-free fetal DNA found in maternal blood offers a promising alternative, though it has not yet reached the status of a definitive diagnostic method. Other non-invasive screening attempts, such as analyzing specific markers in maternal urine or isolating fetal cells from maternal blood, have faced technical challenges and have not gained widespread acceptance in clinical practice. For instance, early studies on urinary biochemical markers of T21, like total estriol, human chorionic gonadotropin (hCG), β-core hCG, and more recently, hyperglycosylated hCG, showed potential, with β-core hCG being notably raised in T21 pregnancies in a multicenter study [16]. However, there was a significant overlap in the biomarker distribution between affected and unaffected patients. Additionally, recent efforts to identify fetal cells in maternal blood and facilitate a non-invasive diagnosis for common chromosomal abnormalities through techniques, like fluorescent in situ hybridization (FISH) or quantitative fluorescence polymerase chain reaction (QF-PCR), have yielded limited results [17,18].

The most common methodology used to detect T21 [19] includes both non-invasive and invasive stages. The first one, having a screening value and providing only a risk assessment, includes biochemical prenatal screening markers, a nuchal translucency measurement via ultrasound, and NIPT. Invasive prenatal diagnostic procedures, which serve as diagnostic tools, include chorionic villus sampling (CVS), amniocentesis, and umbilical cord blood sampling. Although NIPT was introduced commercially as an alternative to traditional invasive testing procedures and numerous studies have validated its clinical application as a sensitive screening tool for common fetal aneuploidies, NIPT remains a screening tool and not a definitive diagnostic test. Studies, such as the one by Liehr [20], provide valuable insights into the balance between the benefits and limitations of NIPT. Key limitations include inaccurate results in cases of confined placental mosaicism, an underestimated impact of maternal factors (such as obesity, maternal age, or maternal malignancy) and several technical issues, particularly in fetal cell isolation for cell-based NIPT [12]. Further concerns include the lack of data on the efficacy of first-trimester screening (FTS) compared to NIPT, as well as variable false positive and false negative rates of the different NIPT tests. Additionally, NIPT is primarily designed and tested for common aneuploidies, limiting its ability to detect rarer or atypical chromosomal anomalies. These limitations could be addressed or complemented by mass spectrometry techniques as they are able to identify wide ranges of biomarkers and analyze them simultaneously, thus enabling comprehensive screening for a variety of fetal and maternal conditions in a single run. With recent advancements in MS technology, such as single-cell resolution and improved sensitivity, MS could potentially overcome some of the technical limitations seen in current non-invasive techniques.

To gain a comprehensive understanding of the metabolic adaptations associated with T21, it is essential to integrate insights from all levels of omics research, including the genome, transcriptome, proteome, and metabolome. Each of these layers addresses specific research questions and offer unique, distinct perspectives of the complex biological landscape. The advent of mass spectrometry (MS) has significantly transformed the field of omics technologies, driving remarkable advancements in both biological and biomedical research. This evolution is largely attributable to the synergy between MS and various omics disciplines, such as proteomics, metabolomics, and lipidomics, which collectively enhance our understanding of complex biological systems. The application of MS in omics is particularly advantageous, as it enables the precise identification of low-abundance biomarkers across a broad spectrum of protein concentrations. This capability establishes MS as a powerful tool for investigating the metabolic changes associated with T21, ultimately elucidating the fundamental mechanisms at play. While NIPT and other prenatal non-invasive diagnostic procedures are widely available and well-integrated into clinical practice, MS-based techniques are not yet routinely used in prenatal screening. Identifying molecular features that can discriminate T21 cases is crucial for implementing cost-effective, targeted MS-based screening approaches. Despite higher costs and the need for trained professionals, MS techniques have been successfully applied in other clinical settings, such as newborn screening. In these settings, MS is used to quantify amino acids, acylcarnitines, and other metabolites from dried blood spots, enabling the detection of inherited metabolic diseases and demonstrating the cost-effectiveness of MS tools in population-wide screening programs.

This review aims to outline the advancements in prenatal screening for T21, particularly emphasizing the progress made in MS-based studies conducted to date and consolidating their contributions to identifying biomarkers linked to T21 by investigating the molecular changes in maternal biofluids during pregnancies. Rather than focusing on prenatal screening in the conventional sense, we highlight how MS-based proteomic and metabolomic analyses have enhanced our understanding of the maternal systemic response to carrying a T21 fetus. In the concluding section, we perform an enrichment analysis of differentially expressed proteins (DEPs) and metabolites (DEMs) reported in the examined studies, providing insights into the pathway-level alterations associated with T21. These insights are intended to support further research in this area and encourage the development of less invasive prenatal screening methods for T21.

## 2. Mass-Spectrometry-Based Omics for Biomarker Discovery

MS is widely regarded as a leading research and analytical technique employed across various omics disciplines. This method is characterized by its remarkable sensitivity, accuracy, and selectivity, along with an extensive dynamic range. Its effectiveness is significantly enhanced when integrated with separation methods, tandem experiments, and data-driven strategies, resulting in thorough research outcomes [21]. In recent years, substantial progress has been made in MS-based omics, particularly in cancer research, where it is commonly applied to investigate a wide array of chemical and biological compounds.

Proteomics encompass the identification, characterization, and quantification of all proteins present in a cell, tissue, or organism, offering essential insights into protein functions, interactions, and post-translational modifications. The advent of MS-coupled techniques has transformed the study of complex biological mixtures, enabling a thorough proteome analysis. This process includes the identification and quantification of proteins, examination of pathological abundance, determination of primary structures, and detection of post-translational modifications, such as phosphorylation, ubiquitination, and acylation, along with the analysis of protein interactions and subcellular localization. Reversed-phase liquid chromatography (RPLC) is commonly employed for the separation of peptides, proteins, and nucleic acids showing high reproducibility, stability, and efficiency [22]. Studies have demonstrated that hydrophilic interaction liquid chromatography (HILIC) is effective for investigating post-translational modifications at both the peptide and protein levels [21,23,24]. Techniques, such as size exclusion chromatography (SEC) and strong cation exchange chromatography (SCXC), also play crucial roles in the multidimensional separation and enrichment of peptides. Additionally, matrix-assisted laser desorption ionization mass spectrometry (MALDI-MS) is a valuable method for rapid profiling with minimal sample preparation, commonly employed in peptide mass fingerprinting, protein profiling, and an analysis of the post-translational modifications, such as glycosylation and phosphorylation, of peptides and intact proteins. A comprehensive understanding of biological systems requires a multifaceted approach encompassing various levels of investigation, including a transcriptome analysis, mRNA degradation, protein dynamics, post-translational modifications, and the assessment of metabolite concentrations and fluxes. The main objective of metabolomics research is to detect and measure metabolites, which act as markers of a person’s genetic profile and environmental factors. Through the examination of these small molecules, metabolomics offers a comprehensive molecular description of the phenotype of a biological system. This methodology enables scientists to explore metabolic pathways, discover disease biomarkers, and acquire knowledge about physiological and pathological mechanisms. In essence, metabolomics seeks to clarify the intricate relationships among genetics, environment, and metabolism, thereby deepening our comprehension of health and disease. In the fields of metabolomics and lipidomics, MS plays a pivotal role and is increasingly integrated with ion mobility mass spectrometry (IM-MS), in addition to traditional separation methods. The authors strongly endorse the comprehensive review by Paglia et al. [25], which explores IM-MS technologies relevant to metabolomics and lipidomics. Overall, these MS-based approaches allow researchers to understand metabolic pathways, identify biomarkers for diseases, and gain insights into physiological and pathological processes. MS has become an essential instrument in the realm of biomarker discovery, presenting numerous advantages that greatly improve the identification and quantification of potential biomarkers within intricate biological samples. MS holds significant promise for T21 screening by facilitating metabolomics and proteomics to identify specific biomarkers associated with the condition, thus enhancing prenatal screening methods. Analyzing maternal biofluids, especially urine or plasma/serum through MS, provides a non-invasive approach, enabling the detection of a wide range of metabolites that reflect the biochemical status of both the mother and the fetus. The exceptional sensitivity and specificity of MS make it particularly effective for analyzing complex biological samples, including maternal plasma and urine, consequently facilitating the identification of low-abundance biomarkers that are essential for early diagnosis. The relationship between the proteome and metabolome is vital, as proteins, particularly enzymes, regulate metabolite levels. This interplay offers insights into the biochemical pathways affected by conditions like T21.

## 3. Review of MS-Based Biomarker Studies for T21

### 3.1. Approach for Literature Review: Search Strategy and Criteria for Inclusion/Exclusion

The literature screening was performed using two scientific databases: PubMed and Embase, using their hierarchically controlled vocabularies for health sciences, Mesh and Emtree, respectively (Figure S1). Key terms included in the search comprised “down syndrome”, “prenatal diagnosis”, and “mass spectrometry”. This process returned 21 search results from PubMed and 19 from Embase, with 9 articles common to the two databases. The articles were analyzed and summarized, and this process was followed by an extensive search of the referenced and citing articles of all included papers. The final list included 55 articles, with 6 of them consisting of literature review papers. The objectives and key findings of these articles are detailed in Table S1. A comprehensive database of proteins and metabolites relevant to T21 differentiation was compiled from the existing literature for pathway enrichment analysis. Protein functional analysis and additional data are presented in Table S2. Also, Table S3 lists metabolites reported in the literature as differentially expressed in T21 along with data on the functional roles of these molecules. The literature review revealed three types of study methodologies applied in the prenatal screening for T21: i. spectral-based discrimination studies, which analyze the mass spectra fingerprints of the control group versus T21 samples, usually employing pattern recognition software for group differentiation; without the identification of molecules responsible for the differentiation; ii. MS profiling studies where specific molecules—e.g., peptides, proteins and metabolites associated with T21—are identified and reported; usually the biomarker status of these molecules is also investigated; iii. targeted quantification of specific molecules, which generally involves a method-development step for a specific class of molecules that is further applied in distinguishing the T21 status. The studies included are briefly summarized in Table 1 and highlight the primary MS techniques utilized, the biological matrices analyzed, key patient characteristics considered in the research, and the principal findings.

In recent years, there has been a growing focus on mass-spectrometry-based omics approaches, as these techniques have proven to be invaluable for gaining deeper insights into disease mechanisms. This review highlights the variety of MS-based methodologies employed over time for biomarker analysis in T21, encompassing both targeted (e.g., SRM analyses) and untargeted approaches. These techniques, outlined in Table 1, vary significantly in terms of their availability, cost, and feasibility for routine clinical applications. Techniques, such as LC-MS and GC-MS, are particularly suitable for clinical and diagnostic labs. Liquid chromatography coupled with Orbitrap or Q-TOF, for example, has already been successfully employed in newborn screening (NBS) for metabolic disorders, where it has proven to be cost-effective. This growth in MS-based techniques has been supported by evolving global regulatory frameworks, such as the European Union’s Regulation 746/2017, which fosters the shift toward personalized medicine. Major manufacturers now offer certified in vitro diagnostic (IVD) instruments, and a growing number of diagnostic kits for various diseases are becoming commercially available, further supporting the potential for these technologies to become a part of routine clinical practice. Its potential use for T21 and other aneuploid screening shows promise, with LC-MS/MS offering an accessible technology for routine clinical testing once properly validated. In this regard, the present paper shows the body of evidence from research studies towards this first step—finding the biomarkers. In context of T21, there were several studies implementing methodologies for biomarker confirmation, ELISA or bead-based multiplexed immunoassays [36,37,40], or directed to assays developed for biomarker quantitation, LC-MS/MS and SRM assays [34,35,38], from various biological matrices.

### 3.2. Proteomic Signature of Prenatal Down Syndrome

In the past decade, proteomics research has made significant advances in the identification of novel biomarkers that can enhance the prenatal diagnosis of T21. This research has primarily focused on the analysis of various biological samples, including amniotic fluid cells (AFCs), amniotic fluid supernatants (AFSs), and serum and urine samples. A notable trend in this field has been the increasing emphasis on less invasive or minimally invasive techniques, which have gathered considerable attention due to their potential to reduce the risks associated with established, more invasive procedures.

#### 3.2.1. Proteomic Analysis of Down Syndrome Amniotic Fluid Samples

The discovery of biomarkers using proteomic techniques has played a crucial role in enhancing prenatal diagnostics for T21. For example, research has revealed changes in the concentrations of proteins in amniotic fluid that are associated with the presence of T21, thus laying the groundwork for the development of diagnostic tests. Several studies published between 2004 and 2021 analyzed the amniotic fluid (AF) proteome in T21 pregnancies compared to those with a normal karyotype (Table 1 and Table S1). One of the first studies using MS-based proteomics in T21 prenatal screening was reported by Oh J. et al. [26]. The authors used 2-DE protein separation followed by in-gel digestion and the MALDI-MS identification of resulting peptides for the proteome profiling of AFC. The AFC samples (*n* = 7) obtained from amniocentesis were collected from patients in the early second trimester of pregnancy (16–18th gestational week), including three T21-positive and four controls. A total number of 99 proteins were unambiguously identified using MALDI-MS and categorized as enzymes implicated in the metabolism of carbohydrates, nucleotides and amino acids and derivatives. In addition, the study reported several DEPs in the amnion cells of patients with T21.

The Diamandis group published several articles focusing on a proteomics analysis of AF in T21. In a paper published in 2013 [27], the authors studied the modifications of proteome profiles in T21 and euploid amniocytes, using stable isotope labeling with amino acids in cell culture (SILAC). A total of 904 differentiating proteins were found using SILAC. Ingenuity pathways analysis (IPA) analysis revealed that the DEPs were involved in pathways such as infection mechanism, cellular assembly and organization, cardiovascular diseases, cell morphology, hematological system development and function, humoral immune response, lipid metabolism, and organismal development [27]. Nine biomarkers were SRM-validated (namely AKAP12, IGF2R, LCRMP, MCAM, NES, PLOD2, PYGL, SOD1 and TPM2). Of these, only two (NES and SOD1) showed differential expression with elevated SOD1 expression and a marked decrease of NES in T21 amniocytes.

Liu et al. [28] reported 41 proteins differentially expressed between T21 and normal amniocytes using 2DE coupled with MALDI-TOF MS. The authors successfully identified calumenin (CALU), nucleophosmin (NPM), elongation factor 1-beta (EF1B2), cathepsin D (CTSD), platelet-activating factor acetylhydrolase IB subunit beta (PAFAH1B2), and 14-3-3 protein beta/alpha proteins, as key proteins. Furthermore, Western blotting analysis confirmed the elevated levels of NPM and CTSD in T21 amniocytes. Tsangaris et al. [29] applied 2-DE coupled with MALDI-MS analysis and nano-ESI-MS/MS to identify proteins differentially expressed in the AFS of pregnancies with T21 fetuses (17 gestational weeks; normal karyotype, *n* = 12; T21, *n* = 6). Five proteins had significantly different levels in the two groups with one uniquely present in T21. The authors confirmed, through Western blotting, the reduction of IBP-1 levels in the AFS obtained from T21 cases. Another study published in 2009 [30] used 2D chromatography separation and MALDI-MS analysis to identify proteins with differential expression in T21 and ES fetuses compared to chromosomally normal ones. The group reported increased levels of antitrypsin (SERPINA3), prealbumin (transthyretin, TTR), and transferrin (TF) in T21, while apolipoprotein A1 was decreased. Western blot and ELISA were used to confirm protein expression. Park et al. [31] applied affinity chromatography and LC-ESI MS/MS for a protein profile comparison of AFS from pregnancies with T21 fetuses and chromosomally normal fetuses; 88 proteins were identified using this approach, 6 were exclusively found in T21 cases, while 11 were specific to the chromosomally normal group; 44 proteins were assessed as differentially expressed between the two groups. Several proteins that play a crucial role in the physiological processes of pregnancy were emphasized, including decorin, hemopexin, and the proform of proteoglycan-2. An important reduction in alpha-fetoprotein (AFP) and apolipoprotein A-II in T21 AFS levels was validated through Western blot analysis.

The Diamandis group [59] developed a bottom-up 2D fractionation strategy involving strong cation-exchange followed by reverse-phase LC fractionation and an MS/MS analysis of the normal AF proteome. The method was applied in a later study [32] to compare the proteome of normal and T21 AFSs. The proteome analysis utilized a pool of ten biological replicates for each group identifying a total of 542 proteins with 396 shared between the two groups. A comparison of the normal and T21-affected AF proteome revealed 181 proteins with differential expression between the groups; the list was further reduced to 60 potential biomarker proteins based on selectivity of the protein to one group, spectra count cutoffs, the fold change, and a cellular component analysis. Amyloid precursor protein (APP) and tenascin-C (TNC-C) were selected for validation using ELISA performed on the same sample type. T21 AFS exhibited high levels of APP but low levels of TNC-C. Unfortunately, these results were not confirmed in maternal serum samples. The study also focused on mapping DEPs to canonical and functional pathways: IPA analysis revealed organ morphology and reproductive system development and function as top pathways associated with the DEPs observed in T21.

In a follow-up paper published in 2011, the Diamandis group [33] developed and applied an SRM assay for the differential expression analysis of thirteen candidate proteins in AF. The study emphasized a significant increase in matrix metalloproteinase-2 (MMP2) and decrease in bile salt-activated lipase (CEL), mucin-13 (MUC13), dipeptidyl peptidase 4 (DPP4), and carboxypeptidase A1 (CPA1) in T21. The MMP2 level was evaluated in maternal serum samples using ELISA; promising AF results were not confirmed in maternal serum. In the following year, the group published SRM assay results applied to 54 AF samples [35]. Seven candidate proteins were selected: chloride channel accessory 1 (CLCA1), hyaluronan and proteoglycan link protein 1 (HAPLN1), mucin 5AC (MUC5AC), PLUNC (palate, lung and nasal epithelium associated), bile salt-activated lipase (CEL), carboxypeptidase A1 (CPA1), and mucin 13 (MUC13). Statistically significant differences were found in six of the seven proteins analyzed with CEL, CPA1, MUC13, CLCA1, and MUC5AC showing significant decreases in T21 AF, while HAPLN1 exhibited a significant increase.

#### 3.2.2. Proteomic Analysis of Down Syndrome Maternal Serum Samples

Biomarker discovery in maternal serum plays a fundamental role in the early diagnosis of T21. In 2005, Busch et al. [60] employed a SELDI-TOF-MS–based ProteinChip^®^ System and bioinformatics to analyze the proteome profiles of maternal serum in T21 fetuses and normal pregnancies implementing a modified fuzzy c-means clustering algorithm to categorize the protein expression levels. The study analyzed serum samples from first-trimester pregnancies. Although specific proteins were not identified, the study reported a prediction accuracy of 91% for T21 pregnancies and 97% for unaffected pregnancies, based on the specific proteomic signatures described.

A 2009 study [36] implemented a bead-based multiplexed immunoassay to identify new potential T21 biomarkers in maternal serum; 90 different analytes were determined using this assay in the serum samples from 14 women carrying fetuses with T21 and 15 women with unaffected pregnancies (both in the first trimester). Alpha-fetoprotein, epidermal growth factor, extracellular receptor-binding protein, insulin, lipoprotein A, eotaxin, and haptoglobin expressed a significant fold change between the groups. While none of the biomarkers defined T21 independently, combining them with established screening markers—PAPP-A, free β-hCG, and nuchal translucency (NT)—improved the overall predictive accuracy. The same research team expanded on their earlier work, analyzing the seven biomarkers previously reported within a larger sample size [37] The study confirmed a significant difference for epidermal growth factor (EGF) expression in T21 alone. The addition of EGF to the existing screening markers resulted in a 5% false-positive rate when differentiating between T21 and normal karyotype pregnancies.

Lopez et al. [38] analyzed serum samples to identify new biomarkers for a first trimester T21 diagnosis using high-resolution LC-MS/MS, with CID and HCD fragmentation modes and an Orbitrap mass analyzer. A selection of the biomarkers with the highest potential was performed based on receiver operating characteristic (ROC) curve analysis; the resulting subset of proteins were used for the development of SRM assays. The analysis revealed 12 proteins with reduced expression levels in T21, including apolipoproteins, their precursors, the enzyme paraoxonase 1, and glycoproteins, such as the histidine-rich glycoprotein precursor and pregnancy-zone protein. IPA associated the identified proteins with antigen presentation and humoral and cell-mediated immune responses, the development of the cardiovascular system, lipid metabolism and cellular development, and growth and proliferation. In a study from 2011, Mastricci et al. [39] identified eight potential serum biomarkers for T21 prenatal screening, including afamin, apolipoprotein E, clusterin, ceruloplasmin, transthyretin, fetuin-A, pigment epithelium-derived factor glycoprotein, and epidermal growth factor). Serum samples from 25 T21 pregnancies and 50 euploid pregnancies (first trimester) were analyzed using ELISA; however, no significant differences were found for any of the proteins selected between the study groups. A year later, a study conducted by Yu et al. [40] employed 2D gel electrophoresis and MALDI-MS for protein identification using serum samples from six T21 pregnancies and six pregnancies carrying fetuses with normal karyotypes; 29 DEPs were reported, with 14 proteins being upregulated, such as serotransferrin, alpha-1b-glycoprotein, desmin, alpha-1-antitrypsin, complement factor B, and ceruloplasmin. Conversely, 15 proteins were downregulated, including serum amyloid P-component, in the T21 group.

A separate study published the same year [61] employed iTRAQ immuno-depletion and LC/MS/MS analysis based on 18 paired serum samples comparing T21 pregnancies with healthy ones. The study identified 31 proteins, with 16 upregulated and 15 downregulated, 18 of which were also reported in previous research. Key potential biomarkers for T21 included alpha-2-macroglobulin, apolipoprotein A1, apolipoprotein E, complement C1s subcomponent, complement component 5, as well as alpha and beta polypeptides, and fibronectin.

Narasimhan et al. [62] conducted a comprehensive proteomic analysis of 23 trisomy cases (T21, T18, and T13) and 85 normal pregnancies during the early second trimester. Using an H50 (C8 reversed phase/hydrophobic interaction chromatography) ProteinChip™ Array and SELDI-TOF-MS, 12 features were identified that could discriminate all trisomies from euploid pregnancies. Unique biomarkers for each trisomy type were also reported. SDS-PAGE coupled with a MALDI TOF/TOF technique emphasized 21 proteins linked to amyloidosis (such as apolipoprotein and transthyretin), acute-phase reactant proteins (including ceruloplasmin and haptoglobin), proteinase inhibitors (like A1AT), and proteins related to neuromuscular development. WB confirmed the differential expression, between trisomic and control serum samples, of four proteins—TTHY, A1AT, apolipoprotein E (APOE), and APOH. Notably, the analysis revealed distinct subunit expression patterns for these proteins in the serum of mothers carrying fetuses with either euploid or trisomic conditions. The 100 kDa subunit of alpha-1-antitrypsin (A1AT) was not detected in cases of T13, T18, and euploid controls but exclusively found in the serum of pregnancies with T21 fetuses. The full-length 55 kDa transthyretin (TTHY) was absent in all tested samples of T13 and T18, and most of the T21 samples but were present in controls. The 35 kDa subunit of APOE was more reliably detected in T18 and T21 and absent in T13 and controls. These specific protein subunit patterns suggest their potential as biomarkers for distinguishing trisomy pregnancies from euploid ones, offering a valuable tool for early prenatal screening.

In a 2015 study, Yao et al. [63] aimed to identify noninvasive, yet predictive, biomarkers for the prenatal diagnosis of T21. For the initial screening, pooled serum samples were collected from five pregnant women in their second trimester, with half carrying fetuses diagnosed with T21. The most notable DEPs, totaling eight, included GTPase, factor B serine protease domain (FBSP), b2-GPI, CFHR1, kininogen 1 isoform 2, complement receptor of the immunoglobulin superfamily (CRIG) bound to C3c, human complement component C3c (HC3c), and human apolipoprotein A-I. WB was conducted on a separate cohort of pregnant women in their second trimester (*n* = 12) to validate the reported proteins. Notably, three protein biomarkers—dGTPase, CFHR1, and kininogen 1 isoform 2—showed significantly higher expression, whereas b2-GPI exhibited lower levels in the serum of pregnant women affected by T21.

The research conducted by López Uriarte et al. [64] aimed to explore and compare the proteomic profiles of serum from pregnant women in their second trimester carrying fetuses diagnosed with T21. The analysis involved blood serum samples from three distinct groups: 10 non-pregnant women, 10 pregnant women with healthy fetuses, and 9 pregnant women carrying fetuses diagnosed with T21. An UHPLC coupled with Q-TOF-MS was used to identify potential new biomarkers. The group reported 10 proteins uniquely present in the T21 group, linked to coagulation and complement pathways, immune responses, and the metabolism of hemoglobin and union proteins. Notably, proteins linked to hematological and immune pathways were highlighted: C4b-binding protein alpha chain (C4BPA) and complement factor H (CFH), both acting as activators of complement pathways, with elevated levels observed in pregnancy-related conditions, such as preeclampsia. Altered complement activation may contribute to conditions, like preeclampsia and gestational diabetes; alpha-1 acid glycoprotein (A1BG) increases in response to inflammation and is elevated in pregnancy complications. Beta-2-glycoprotein (β2-GPI) is involved in coagulation and angiogenesis; also, it is linked to recurrent miscarriage and first-trimester pregnancy loss. Both melanotransferrin (MELTF) and hemopexin (HPX) are involved in heme and iron metabolism with abnormal expression correlated with miscarriage. Two proteins particularly relevant in the context of T21 were emphasized, namely apolipoprotein E (ApoE) known for its involvement in Alzheimer’s disease (more prevalent in individuals with T21) and Kininogen 1 (KNG1)—implicated in the development of nuchal translucency, a marker for chromosomal abnormalities in T21.

#### 3.2.3. Proteomic Analysis of Down Syndrome Maternal Plasma Samples

The initial proteomic investigation aimed at identifying novel biomarkers for the prenatal screening of T21 was conducted by Kolialexi et al. [41] and published in 2008. The group reported three serum carrier proteins significantly elevated in pregnancies affected by T21, namely transthyretin (TTR), ceruloplasmin (CP), and afamin (AFM). Additionally, an acute-phase protein (the precursor of alpha-1-antitrypsin), as well as precursors of apolipoprotein E, serum amyloid P-component, AMBP protein, and histidine-rich glycoprotein, were also found to be significantly upregulated. Conversely, the clusterin precursor, another acute-phase protein, was noted to have lower expression in T21 cases.

In a separate investigation, iTRAQ labeling combined with MALDI/TOF/TOF was employed to discover novel protein biomarkers for T21 screening [42]. A limited set of six paired samples, consisting of both T21 and normal pregnancies, was examined, revealing 178 proteins with differential expression between the two groups. Among these, 28 proteins were found to be significantly upregulated, which included immunoglobulins, complement components, enzymes, and signaling and carrier proteins. Conversely, 13 proteins exhibited downregulation, comprising extracellular matrix glycoproteins, transport proteins, such as afamin, peptide hormones, like apolipoprotein (a), and proteins associated with cell adhesion, including von Willebrand factor, lumican, and heparan sulfate proteoglycan.

Heywood et al. [43] conducted an extensive investigation into maternal plasma proteome to identify potential biomarkers for T21 using various techniques. Initially, they studied 2D-DIGE with three distinct pH ranges to examine plasma samples from paired T21 and normal pregnancies during the first and second trimesters. While no significant differences were found between first trimester pregnancies, in the second trimester (pH range 4.5–5.5), seven proteins were significantly upregulated in T21 cases, including ceruloplasmin (CP), inter-alpha trypsin inhibitor heavy chain H4 (ITIH4), complement C4-A and C5 precursors, complement component C9 precursor, kininogen-1 (KNG1), and complement C1s subcomponent precursor. Additionally, the team developed a quantitative method [46] for detecting serum amyloid-P and C1-inhibitor protein using SRM UPLC-MS/MS, reporting elevated levels of these biomarkers for 10–14 weeks and 14–20 weeks gestation.

In a follow-up study, Heywood et al. [45] studied the dynamics of the maternal plasma proteome by analyzing samples across two gestational age ranges: 10–14 weeks and 14–20 weeks, including both T21 and normal pregnancies. A screen for differentially expressed proteins was conducted using SELDI-TOF-MS. Initial testing involved eight pairs of plasma samples from T21 and control subjects and employed four distinct ProteinChip arrays: Q10 (SAX), CM10 (SCX), hydrophobic (H50), and immobilized-metal ion (IMAC). Notable differences in protein expression were detected exclusively with the SAX array, which was subsequently used for the analysis of the remaining samples. In the 10–14-week group, plasma protease C1-inhibitor was significantly elevated, while in the 14–20-week group, serum amyloid P-component and transthyretin levels increased and complement C3-α chain decreased. Validation via SDS-PAGE and Western blotting confirmed the findings and, particularly for transthyretin, the mass changes correlated with both the gestational age and T21 were noted as being attributed to the protein’s post-translational modifications.

Sui W. et al. [46] employed iTRAQ, strong cation exchange (SCX) chromatography, and MS/MS to explore protein profile alterations in the umbilical cord blood associated with T21. They identified 505 proteins, with 19 being differentially expressed. The authors focused on five specific DEPs—apolipoprotein E, complement factor B (CFB), acute-phase C-reactive protein (APCS), matrin-3, and osteopontin (OPN)—highlighting their potential as biomarkers for T21.

#### 3.2.4. Proteomic Analysis of Down Syndrome Maternal Urine Samples

Shan et al. [65] used MALDI-TOF-MS with magnetic beads (MB-WCX) for peptide fractionation from concentrated maternal urine samples to identify biomarkers for the prenatal screening of T21. They focused on comparing peptides between women carrying T21 fetuses and those with normal karyotypes between 17–22 weeks of gestation. Six peptides were significantly different, including parts of alpha-1-antitrypsin (SERPINA1) and heat shock protein beta-1 (HSPB1). Similarly, Iles et al. [66] analyzed urine samples from women at 12–17 weeks using MALDI-TOF-MS, observing that T21 samples showed distinct spectral peaks: the T21 group exhibited additional spectral peaks between 11,000 and 12,000 *m*/*z* and less intense peaks in the 6000–8000 *m*/*z* range being possible to distinguish between T21 fetuses and controls at 12–14 weeks.

These studies highlight the potential of urine peptide profiling as a non-invasive approach for early T21 screening.

### 3.3. Metabolomic Signature of Prenatal Down Syndrome

The integration of analytical platforms, like LC-MS, GC-MS, and CE-MS, has significantly advanced metabolomics, expanding the range of detectable metabolites. Each platform has unique strengths and limitations, with the choice depending on factors, such as the metabolite type, sample, and the need for sensitivity or high throughput. The continuous improvement of these techniques enhances metabolomics’ role in biomarker discovery and disease screening, especially with non-invasive samples, like urine, for clinical use.

#### 3.3.1. Metabolomic Analysis of Down Syndrome Amniotic Fluid Samples

MS-based metabolomics techniques have proven to be powerful for investigating altered metabolic pathways in diseases like T21. In the context of T21, several studies have focused on analyzing AF from pregnant women carrying fetuses with T21, as AF is rich in fetal metabolites and can offer valuable insights into fetal development and disease-associated metabolic changes. Baggot et al. [48] employed GC-MS to analyze the organic acid profiles of AF samples from T21 and normal karyotypes. They found significant increases in organic acids, such as 5-hydroxycaproate, methyl succinate, α-ketoglutarate, adipate, and phenylpyruvate, linking these to disruptions in tetrahydrobiopterin metabolism and potential vitamin B2 deficiency in T21. Huang et al. [49] used LC-employed LC-MS for metabolomics of amniotic fluid samples from women undergoing amniocentesis during the second trimester. The report utilized two complementary stationary phases for metabolite profiling: hydrophilic interaction liquid chromatography (HILIC) and reverse phase chromatography, studying both positive and negative ionization conditions. Significantly different features in T21 were identified: notably, hydrocortisone and L-glutamine levels were significantly elevated in T21, while coproporphyrin-III, L-glutamate, pregnenolone sulfate, taurochenodeoxycholate, L-arginine, taurocholate, and L-histidine and glycocholic acid were significantly reduced. Pathway analysis revealed alterations in amino acid metabolism, liver function, growth hormone regulation, and neural development, along with significant changes in pathways related to chromosome 21 genes, including galactose metabolism, purine metabolism, and neurodegenerative disease pathways.

Similarly, Liu et al. [50] used HILIC and reverse-phase chromatography (RP) coupled with UPLC-MS/MS to analyze AF from T21 cases and controls. This strategy identified 621 compounds, with 151 metabolites showing significant differences between the two groups. Hierarchical clustering and random forest analysis were used to categorize the metabolites important for group classification into four main metabolic pathways: gamma-glutamyl amino acids, steroid hormone derivatives (notably reduced in T21), and polyamines and glycerol derivatives resulting from phospholipid degradation (elevated in T21). Additional altered pathways included gamma-glutamyl amino acids, phospholipid metabolism, fatty acid metabolism, and pentose metabolism.

A separate study [52] examined the metabolomic profiles of samples from amniocentesis performed between the 15th and 18th weeks of gestation, including 13 women with fetuses diagnosed with T21 and another consisting of 13 women with healthy pregnancies. A reverse phase separation technique combined with a Q-TOF MS detector operating in both positive and negative modes was employed for the analysis. Consistent with earlier findings, an increase in diacetyl spermine was observed in pregnancies affected by T21. Additionally, three metabolites—p-cresol sulfate, methylhistidine, and hexanoylcarnitine—were significantly decreased in the AF of T21-affected pregnancies. These alterations suggest potential disruptions in nervous and muscular system development associated with T21. In another investigation, Chen et al. [51] aimed to identify altered metabolic pathways in T21 and potential biomarkers for screening by analyzing both urine and AF samples. Their analysis of AF identified 351 metabolites that effectively differentiated T21 from control profiles, with many linked to carbon metabolism, the TCA cycle, organic acid metabolism, and glucagon signaling. The most enriched pathways involved D-glutamine and D-glutamate metabolism, as well as alanine, aspartate, and glutamate metabolism. These compounds are critical for nerve development and the oxidative stress response. In contrast, the number of unique metabolites found in urine was lower, primarily associated with phenylalanine metabolism.

#### 3.3.2. Metabolomic Analysis of Down Syndrome Maternal Plasma and Serum Samples

Metabolomics is an emerging field that offers a comprehensive snapshot of metabolic changes occurring in the body. By profiling metabolites in biological fluids, like plasma and serum, researchers may uncover disrupted metabolic pathways indicative of certain conditions, including pregnancy complications and fetal diseases. The analysis of metabolites using maternal plasma and serum represents a powerful approach for understanding the interplay between maternal and fetal health during pregnancy. Maternal metabolic changes not only reflect the health status of the mother but also offer valuable clues regarding potential fetal conditions. Parfieniuk et al. [54] highlights this connection, showing how maternal plasma metabolomics can be used to identify metabolic alterations associated with various fetal conditions, including T21.

Singh et al. [53] used NMR-based metabolomics to examine maternal serum samples from the first trimester of pregnancy comprising 30 T21 cases and 60 controls. Notably, three novel maternal serum upregulated metabolites (3-hydroxybutyrate, 3-hydroxyisovalerate, and 2-hydroxybutyrate) emerged as the most significant discriminators. These metabolites are crucial for phospholipid and cerebroside synthesis, important for myelination and brain development, processes impaired in T21. 3-Hydroxyisovalerate is typically excreted in urine and serves as a biomarker for biotinidase deficiency, which clinically manifests as hypotonia, learning disabilities, seizure disorders, and brain atrophy. Additionally, 2-hydroxybutyrate, an organic acid, is found to be elevated during oxidative stress, potentially harming neonatal brain development.

In 2018, Parfieniuk et al. [54] aimed at identifying novel metabolites that could serve as biomarkers in maternal plasma samples from pregnancies affected by T21 (T21 = 12; control = 15). UPLC-QTOF-MS operated in both positive and negative modes was used for analysis. Five metabolites were found to be significantly downregulated in the T21 samples: three fatty acids (palmitic amide, linoleamide, oleamide), butyryl-L-carnitine, and piperine, all being linked to the development of the fetal nervous system.

In a study conducted by Nemutlu et al. [55], plasma samples were examined through both GC-MS and LC-qTOF-MS techniques, uncovering unique metabolomic profiles that differentiate pregnant women expecting fetuses with T21 from those with healthy fetuses. GC-MS metabolomic profiling revealed notable differences in the levels of several metabolites in pregnancies affected by T21, including the upregulation of 2-hydroxybutyric acid, benzoic acid, nonanoic acid, 3-hydroxybutyric acid, and 2-ketoisocaproic acid and downregulation of threonic acid, beta-alanine, creatinine, alpha-tocopherol, pyruvic acid, cholesterol, uracil, oxalic acid, 2-piperidone, and isopropyl benzoic acid. Additionally, LC-qTOF-MS demonstrated significant changes in the concentrations of creatine, N4-phosphoagmatine, citrate, 2,5-dioxopentanoate, 2-furoate, pyruvate, fructose, and various lipid-related metabolites. The authors attributed the 2-hydroxybutyric acid elevation to a compensatory response to the deficiency in glutathione synthesis caused by increased oxidative stress in the liver. As 2-hydroxybutyric acid results from the catabolism of L-threonine, implicated in DNA methylation, the reported findings suggest that reduced levels of threonine could contribute to associated physiological and pathological events in T21. These altered pathways, together with impaired folate metabolism may play a role in the development of congenital malformations and aneuploidies [67,68]. 2-Hydroxybutyric acid can be increased in conditions of deficiencies of energy metabolism [69]. 3-Hydroxybutyrate, a ketone produced in the liver from acetyl-CoA, serves as an alternative energy source for the brain during hypoglycemic episodes and supports brain development and myelin production. Increased levels of this metabolite in the maternal plasma of pregnancies affected by T21 may be linked to the altered myelin formation and cortical dysgenesis seen in children with T21, contributing to neurocognitive delays and growth issues. Beta-alanine, known for its antioxidant properties and role in muscular endurance, may also be compromised in T21. Additionally, this study revealed altered vitamin C metabolism in pregnancies with T21 fetuses sustained by the decreased levels of L-threonic acid reported in this study. Furthermore, a decrease in alpha-tocopherol has been reported; its deficiency is linked to neurodegenerative diseases. Uracil, downregulated in T21, is involved in DNA repair and neuroprotection, with its reduction possibly reflecting a compensatory response to T21-associated neurodegeneration [55].

Based on the results previously described in a study and the literature, Özkan et al. [56] developed a GC-MS method for the simultaneous identification of 2-hydroxybutyric acid, 3-hydroxybutyric acid, β-hydroxyisovaleric acid, uracil, glutamic acid, maltose, and melezitose. The method was applied to plasma samples from 22 pregnant women carrying T21 fetuses and 11 carrying euploid fetuses. The results showed that all seven metabolites were significantly elevated in the plasma of women with T21 pregnancies, linking them to key metabolic pathways, such as ketone body metabolism, amino acid metabolism, and nucleotide metabolism, which are crucial for energy production and neurodevelopment. In the subsequent study, Özkan et al. [57] used LC-MS to quantitatively analyze 3-hydroxybutyric acid and 3-hydroxyisovaleric acid, focusing on a polar stationary phase for improved separation. The validated LC-MS method was applied to 17 plasma samples from pregnant women carrying T21 fetuses and 30 from women carrying euploid fetuses. Consistent with the GC-MS study, elevated levels of both 3-hydroxybutyric acid and 3-hydroxyisovaleric acid were observed in T21 pregnancies, suggesting alterations in energy metabolism, particularly in the synthesis of ketone bodies and branched-chain amino acids.

#### 3.3.3. Metabolomic Analysis of Down Syndrome Maternal Urine Samples

Urine metabolomics is an emerging, non-invasive method for the prenatal screening of T21, offering significant potential for biomarker discovery. Urine, as a biofluid, contains a wide range of metabolites reflecting systemic metabolic changes that occur throughout pregnancy (involving the mother’s physiology, placental function and fetal growth). Additionally, the metabolites present in urine reflect both maternal and fetal metabolic processes, making it a valuable resource for identifying biomarkers related to pregnancy complications and fetal development.

The research conducted by Trivedi and Iles [58,70] underlines maternal urine as a valuable and non-invasive resource for metabolomic studies demonstrating its viability for prenatal screening. In their studies, the authors established a comprehensive baseline of normal metabolic changes occurring during pregnancy [70] and identified potential features for pathological pregnancies, including T21 [58]. The researchers analyzed urine samples collected from both normal and pathological pregnancies (including those carrying T21 fetuses) between 9 and 23 weeks of gestation, encompassing both the first and second trimesters. In total, 383 features were analyzed revealing significant changes in several features after the 14th week of gestation associated with the development of a distinct metabolic fingerprint as pregnancy progresses, likely in response to the evolving metabolic requirements of both the mother and the fetus.

In a subsequent study, the Iles group [58] expanded their work on maternal urine metabolomic profiles to identify abnormal fetal markers associated with T21 and other trisomy cases. The study included 122 maternal urine samples, comprising 93 normal pregnancies, 23 T21 pregnancies, and 8 pregnancies with other aneuploidies. The methodology applied in this study included both HILIC and RP chromatography coupled with MS for better coverage of urine metabolites. Partial least square discriminant analysis (PLS-DA) performed on HILIC-obtained metabolomic profiles revealed that the T21 urine samples can be distinctively separated from the normal cases. Notably, the model achieved 0% false positive results and 60.87% accuracy for identifying T21 cases. The urine samples from unknown trisomy cases exhibited a similar metabolic profile to T21. Moreover, SIMCA P + modeling based on the measured ion profiles was able to accurately classify (100%) the normal urine samples and 87% of the T21 cases when T21 and other trisomy cases were treated as separate groups. The authors conducted structural elucidation and analyte identification for the masses revealed as promising features based on the multivariate analysis, being able to report dihydrouracil (*m*/*z* 114.07) and progesterone (*m*/*z* 314.20) as key metabolites with changes in the maternal urine that significantly coincided with the presence of a T21 fetus. Dihydropyrimidinase enzyme deficiency, linked to reported elevated levels of dihydrouracil closely resemble T21 symptoms, such as developmental delays and facial dysmorphism. Additionally, urinary progesterone levels were consistently low in all T21 samples, indicating reduced feto-placental unit activity and lower excretion of progesterone and its metabolite, 5α-dihydroprogesteron. These findings suggest that altered progesterone metabolism may be linked to T21 pregnancies and may reflect underlying feto-placental dysfunction.

## 4. Assessment of Modified Pathways and Their Importance in Down Syndrome Pathology—A Sample-Type-Based Assessment Using Differentially Expressed Proteins Reported in the Literature

A functional analysis of DEPs reported in the literature for a specific disease can provide valuable insights allowing for deeper insights into the underlying biological mechanisms and identification of pathway alterations and can highlight potential biomarkers or therapeutic targets for further investigation. However, there are both strengths and challenges associated with this approach. On the one hand, the approach builds on the existing body of research, using previously reported DEPs that have already been implicated in T21 providing a holistic view of how various altered proteins work together within the biological network of disease. Pathway enrichment analysis and the functional annotation of DEPs can highlight key biological pathways and processes that are or important molecular mechanisms that drive the pathology, such as cell cycle regulation, neurodevelopment, the immune response, or metabolic disorders. On the other hand, literature-derived data may be heterogeneous being highly impacted by the different sample types, experimental conditions, and methodologies implemented. Another point is that not all DEPs reported in the literature may be well-characterized or fully annotated. Additionally, the present study is limited by a lack of experimental validation as this analysis is computational or bioinformatics-based. Despite these limitations, the authors believe that leveraging existing data is a valuable strategy for advancing our understanding of T21, acknowledging the need for further experimental validation and the potential biases inherent to this approach.

### 4.1. Differential Expression of Proteins and Metabolites in Trisomy 21 Across Biological Matrices

**Figure 1:**
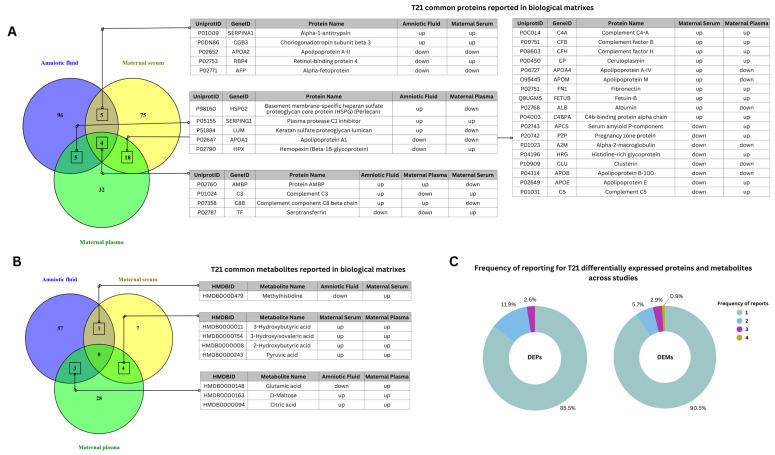
Differentially expressed proteins and metabolites in Trisomy 21 across biological matrices. (**A**) Venn diagrams of common and matrix-specific differentially expressed proteins (DEPs) reported in T21 studies. (**B**) Venn diagrams of common and matrix-specific differentially expressed metabolites (DEMs) reported in T21 studies. (**C**) Frequency of DEPs and DEMs reported in the literature.

A total of 235 DEPs and 100 DEMs associated with T21 were identified from the literature, with mass spectrometry as the primary analytical tool employed (Tables S2 and S3). Figure 1A,B illustrate the common DEPs and DEMs reported across different biological matrices, along with their main direction of expression in T21 compared to control groups. Notably, four proteins with differential expression were reported across all biological matrices included in the study: AMBP, C3, C8B, and TF. These proteins are all associated with complement and coagulation cascades, though they exhibited different expression tendencies across the various matrices. Five proteins were reported in both amniotic fluid and maternal serum, with only one (RBP4) exhibiting differential expression between these matrices. Additionally, five other proteins were commonly reported in both amniotic fluid and maternal plasma. Among them, only two exhibited the same expression trend in T21 across both matrices (SERPING1—higher expression, APOA1—lower expression). Eighteen proteins were commonly reported in the maternal plasma and serum matrices, with most of them showing consistent expression trends in T21 compared to control groups. Specifically, there was a higher expression of complement factors C4A, CFB, CFH, and CP, as well as the acute-phase proteins FT1, FETUB, and C4BPA complement regulatory protein, in T21 samples. In contrast, A2M, CLU, and ApoB proteins were found to have lower expression levels in T21 compared to controls.

No common DEMs were reported across all the matrices studied (Figure 1B). However, one common metabolite, methylhistidine, was identified in both the amniotic fluid and maternal serum, exhibiting different expression levels in these matrices. Additionally, three metabolites—glutamic acid, D-maltose, and citric acid—were commonly reported in both amniotic fluid and maternal plasma. Four DEMs, including 3-hydroxybutyric acid, 3-OH-isovaleric acid, 2-hydroxybutyric acid, and pyruvic acid, showed the same expression trends in T21 maternal plasma and serum.

In Figure 1C, a graphical representation of the frequency of differentially expressed proteins and metabolites reported across T21 studies is included. Among the DEPs, those reported more than twice accounted for 14.5%, while DEMs reported more than twice made up 9.5% of the total identified proteins and metabolites, respectively.

### 4.2. Methodology Implemented for Enrichment Analysis of Literature-Reported Differentially Expressed Proteins

Here, enrichment analysis and visualization were performed using ClueGO [71] (v.2.5.10) and CluePedia [72] (v.1.5.10) within Cytoscape [73] (v.3.10.1). The metabolic pathway implications of the DEPs were investigated utilizing the Reactome database [version 25.05.2022, containing 2535 terms and 10,882 unique genes). Additionally, a term (pathway) was considered enriched if at least three proteins were associated with the term and had a Bonferroni corrected *p*-value of less than 0.005. This analysis encompassed both downregulated and upregulated T21 reported proteins (the complete list is included in Table S2 along with Uniprot [74] extracted data describing protein function). Only proteins reported as significant with a *p*-value cutoff of 0.05 in the original studies were included. Enrichment analysis was performed using the DEPs identified in each biological matrix. A comprehensive list of the pathways derived from the enrichment analysis for each biological matrix and reported regulation in T21 is provided in Table S4. Additionally, graphical representations of the results can be found in Figures S2 and S3. The pathway network representation was organized using the CluePedia option to incorporate all ontology parent terms up to the root node, leveraging the inherent hierarchical structure of the Reactome database and providing enriched context by placing pathways within their broader biological categories. The hierarchical relationship between pathways was visually represented with arrows.

### 4.3. Pathway Analysis of Down Syndrome Differentially Expressed Proteins

**Figure 2:**
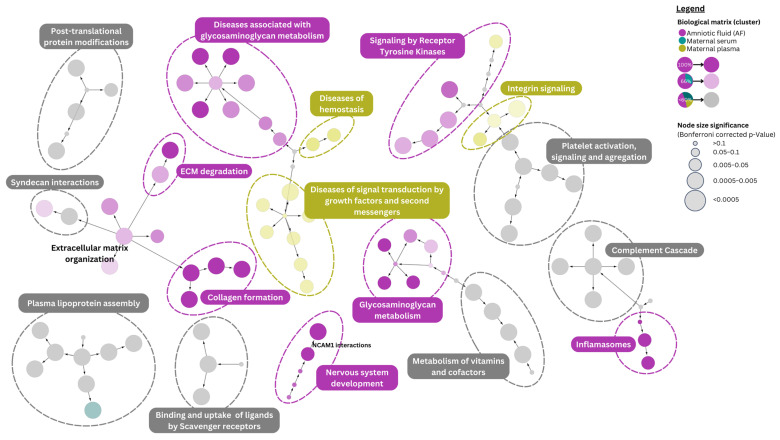
Enrichment analysis of differentially expressed proteins in T21 reported for three different matrices. Bonferroni corrected *p*-value cutoff: 0.005, min 3 genes/term; the node size shows the term significance: the biggest terms are the most significant ones. The node color shows the proportion of genes from each cluster that are associated with the term. Specific terms: >60% genes from a cluster, the node color is based on the proportion of genes and is a gradient white of the cluster color. Common terms: <60%, node color: gray. The hierarchical relationship between pathways was visually represented with arrows. Note on clustering: the clustering in the figure groups pathways according to their parent nodes. While the local distance between clusters is visually presented, it does not represent a specific biological or computational relationship and instead serves to highlight the pathway groupings based on their parent nodes.

This chapter presents a detailed analysis of differentially expressed proteins (DEPs) in T21 across three biological matrices: amniotic fluid (AF), maternal serum, and maternal plasma. A summary of the most significant biological pathways affected across the different T21 matrices is provided in Figure 2. Figures S2 and S3 offer a deeper perspective into the specifics of protein abundance in T21, highlighting DEPs with both higher and lower abundances in the matrices studied.

Several signal transduction pathways were highlighted in the analysis. Notably, DEPs identified in T21 AF were involved in receptor tyrosine kinase signaling, particularly the platelet-derived growth factor (PDGF) and MET pathways (Table S4, Figures S2 and S3). PDGF family proteins play a crucial role in stimulating the growth and motility of connective tissue cells (e.g., fibroblasts, smooth muscle cells), neurons, and capillary endothelial cells by activating tyrosine kinase receptors and initiating signal transduction. PDGF signaling has been linked to transient abnormal myelopoiesis, a preleukemic syndrome that occurs in a subset of T21 newborns. While this condition often resolves spontaneously, in some cases, it can progress to hepatic necrosis and failure. Furthermore, elevated PDGF expression has been observed in the megakaryoblasts of T21 patients with hepatic disease [75]. The MET pathway, which interacts with integrins, such as PTK2—a focal adhesion-associated protein kinase—plays a key role in promoting cell motility. While MET signaling is primarily associated with cancer cell invasiveness, altered PTK2 expression in T21 has been documented, particularly in processes affecting the nervous system and connective tissues [76].

Pathways related to hemostasis, particularly platelet activation and signaling, and the clotting cascade, were observed in all three matrices: AF, maternal serum, and plasma. While the exact role of these affected pathways in the manifestations of T21 remains unclear, emerging evidence suggests a link between platelet activation, signaling, and aggregation and individuals with T21. A study analyzing genes associated with an increased risk of thrombosis in T21 patients found that these individuals have an elevated risk of inherited thrombophilia [77]. Additionally, other case reports have proposed a possible link between T21 and a prothrombotic state [78].

Enrichment analysis highlighted the disruption of integrin signaling, particularly in the maternal plasma of T21 pregnancies. The alteration was most evident in pathways linking integrin signaling to the mitogen-activated protein kinase (MAPK) pathway. Integrins are heterodimeric transmembrane receptors that mediate cell adhesion to the ECM and transmit extracellular signals to intracellular pathways, notably the MAPK pathway. This bidirectional signaling is crucial for regulating key cellular processes, such as proliferation, differentiation, and migration—functions essential for embryonic and placental development. The MAPK pathway plays a vital role in placental development and the formation of the blood–placental barrier [79]. Disruptions in integrin–MAPK signaling in T21 pregnancies may contribute to placental insufficiency, which could be involved in some of the clinical complications observed in these pregnancies.

Other DEPs identified in the AF of T21 cases were involved in neural cell adhesion molecule (NCAM) signaling for neurite outgrowth and NCAM1 interactions. NCAM, a membrane-bound protein from the immunoglobulin superfamily, plays a key role in nervous system development. Disruptions in NCAM pathways have been linked to neurodevelopmental disorders, as seen in T21 [80].

Some DEPs reported in T21 maternal serum samples appear to be involved in signal transduction pathways related to the regulation of NR1H2 and NR1H3, which are linked to cholesterol transport and efflux. NR1H2 and NR1H3 are nuclear receptors found in liver cells, peripheral cells (such as macrophages), and intestinal cells. In the liver, they are activated by ligands (such as oxysterols) and induce the transcription of genes that help protect cells from cholesterol overload. In macrophages, they promote cholesterol efflux, and in the intestine, they reduce cholesterol absorption. Through these mechanisms, NR1H2 and NR1H3 play cardio-protective roles against atherosclerosis, myocardial ischemia, diabetic cardiomyopathy, and myocardial hypertrophy [81]. However, no other correlations between these pathways and T21 pregnancies have been found in the literature.

Completing the proteome signature of T21, DEPs were implicated in extracellular matrix (ECM) organization. Specifically, pathways related to collagen formation and ECM degradation were prominently observed in T21 AF. Additionally, modifications in pathways related to ECM proteoglycans, syndecan interactions, and laminin interactions were most notably affected in T21 AF. Commonly, integrin cell-surface interactions were observed in both T21 AF and maternal plasma (Figures S2 and S3). Non-integrin membrane–ECM interactions were also highlighted in the enrichment analysis. Though less studied, these interactions are crucial for cellular processes and tissue organization. The ECM plays a central role in fetal cardiovascular development, especially in the early stages. Later, there is a transition from a more flexible, less organized ECM to a more structured, collagen-rich ECM. As the cardiac ECM is involved in the structural integrity and function of the cardiac muscle, disturbances in its composition can be associated with congenital anomalies in T21 cases. At the same time, the overexpression of genes coding ECM proteins and proteoglycans was reported in the transcriptional profile of T21 fetuses’ hearts [82,83]. Moreover, several proteomic studies analyzing AF showed an increase in the collagen α-1 (I), α-1 (III) and α-1 (V) chain precursors of type I, III, and V collagen [29,31,59]. While these collagen types are essential components of fibrillar collagen found in various connective tissues, such as bone, tendon, skin, lung, and blood vessels, there was no specific link established in the literature between these collagen types and T21. Notably, the implications of collagen VI α-1 and α-2 chains in some of the pathologies prevalent in T21 patients have been reported, such as congenital heart defects and muscle hypotonia [84,85] or particularities of T21 patients (nuchal skin) [86]. One study included in this review [34] highlighted the increased oxidation of collagen α-2 (I) and collagen α-1 (V) chains in the AF of pregnancies carrying T21 fetuses.

Other major dysregulated pathways in T21 were related to protein metabolism. Specifically, pathways associated with amyloid formation showed lower expression in maternal T21 plasma. Additionally, post-translational protein modifications via phosphorylation were observed across all three matrices. Dysregulation was also visible in pathways regulating insulin-like growth factor (IGF) transport and uptake by insulin-like growth factor binding proteins (IGFBPs), with alterations across all three matrices. Amyloids are fibrillar protein deposits that, when abnormally accumulated, contribute to neurodegenerative diseases, like Alzheimer’s disease (AD). Since individuals with T21 have an increased risk of developing AD, numerous studies have investigated fibrillar β-amyloid peptide deposits in T21 patients [87,88]. These studies highlight an early onset of AD in T21, with β-amyloid plaques appearing around age 40 and progressively increasing with age [87]. β-Amyloid is produced from amyloid precursor protein (APP), which is located on chromosome 21 and overexpressed in T21. Enzymes, such as β-secretase and BACE2, are also involved in β-amyloid production, with BACE2 also located on chromosome 21. Notably, higher levels of BACE2 RNA have been reported in T21 fetal tissue compared to controls [89]. Additionally, studies on T21 fetal brain astrocytes and neuronal cultures have revealed dysfunctions in β-amyloid precursor protein processing [90].

The IGF system plays a crucial role in cell development, growth, and differentiation. It includes the primary growth factors IGF1 and IGF2, their receptors (IGF1R and IGF2R), and IGFBPs, which regulate the IGF bioavailability, activity, half-life, and receptor interactions. Proteases, like pregnancy-associated plasma protein A (PAPP-A), cleave IGFBPs, modulating IGF activity, particularly in fetal growth. While IGF1 and IGF2 genes are located on chromosomes 11 and 12 (not chromosome 21), this signaling pathway is vital for prenatal cell development, apoptosis, and fetal growth. When IGF1 binds to IGF1R, it activates intracellular tyrosine kinase activity, promoting cell proliferation. Lai et al. [91] reported decreased IGF1R and PAPP-A levels in T21, while IGF1 and IGF2 levels remained unchanged between aneuploidy and euploidy cases. These findings suggest that reduced IGF1R expression may contribute to the T21 pathophysiology, particularly by impairing cardiomyocyte proliferation during embryonic development, which could lead to congenital heart defects. Another study reported decreased IGF1 levels in the plasma of T21 patients, with reduced IGF1 signaling linked to inflammation, neurodegeneration, and a short stature [92]. Interestingly, a strong positive correlation was found between the neurofilament light chain (NfL)—a biomarker of neurodegeneration—and IGFBP2, a negative regulator of IGF1 signaling. Conversely, a negative correlation was reported between NfL and IGF1, IGFALS, and IGFBP3, which prolongs IGF1’s half-life [92]. Other neurodegeneration biomarkers, such as UCHL1 and GFAP, were negatively correlated with IGF1 and IGFALS but positively correlated with IGFBP2. Plasma levels of NfL and UCHL1 were also significantly elevated in T21 patients compared to controls [92]. Moreover, the varying effects of IGFBPs on IGF1 levels may explain the presence of both up- and downregulated proteins in T21 AF. Low IGF1 levels in T21 could be attributed to delayed maturation of the IGF system and disturbances in the transition from fetal IGF1 to growth hormone-regulated IGF1 [93].

Modifications in pathways related to the innate immune system were also highlighted by pathway analysis. First, DEPs associated with both maternal serum and plasma were involved in complement cascade pathways. Dysfunctions in these pathways, as part of the innate immune system, may contribute to the immune deficiencies observed in T21 patients. Additionally, complement dysregulation has been identified in plasma samples from T21 patients, with notable differences between T21 individuals with and without AD. Given that complement proteins play a role in β-amyloid plaque formation, their altered expression in T21 could be relevant to the early onset of AD in these patients [61,94]. Furthermore, differential expression of complement proteins may also correlate with chronic infections, inflammatory states, accelerated aging, obesity, and cognitive decline [90]. Second, the NLRP3 inflammasome, a member of the nucleotide-binding domain leucine-rich repeat-containing receptor (NLR) family, plays a crucial role in innate immune responses and was particularly enriched in T21 amniotic fluid samples. NLRP3 activation promotes the release of pro-inflammatory cytokines, including interleukin (IL)-1β and IL-18, and is often implicated in inflammatory and autoimmune diseases [95]. Dysregulation of the immune system, increased susceptibility to infections, and a higher incidence of chronic inflammatory conditions is commonly observed in T21 patients. Studies have shown that individuals with T21 exhibit a distinct inflammatory profile, including elevated levels of pro-inflammatory cytokines, such as IL-1β [96,97].

Plasma lipoprotein assembly, remodeling, and clearance may be disrupted in T21 pregnancies as DEPs reported in both maternal serum and plasma were associated with this pathway. Additionally, DEPs with lower levels reported in T21 maternal serum were associated with plasma lipoprotein clearance, including APOA1, APOE, and APOH, which were identified (see Table S4). APOA1 was previously linked to amyloidosis [62,63], having generally lower expression in T21 plasma and AF when compared to the control group. In contrast, APOE had higher expression in T21 maternal plasma [41,46]. In the context of women with T21 pregnancies, studies highlight the presence of ApoE-ε4 allele and increased levels of blood cholesterol, which impair microcirculation, including in the vessels surrounding the ovarian follicle, with negative consequences on meiotic division and fertility [98,99]. DEPs involved in ligand binding and uptake by scavenger receptors were identified across all matrices in our pathway analysis. Scavenger receptors have multiple roles in physiological processes, including the immune response, oxidized LDL clearance, and bacterial elimination. They are expressed in tissues and cell types, such as the macrophages, lungs, placenta, intestines, heart, and epithelial cells, where they help protect against reactive oxygen species (ROS), facilitate oxidized LDL degradation, and support bacterial clearance from the lungs, bloodstream, and epithelial surfaces [100].

The pathway analysis of the literature-reported DEPs revealed several significant metabolic alterations in T21, particularly in the retinoid metabolism and transport pathway. Retinoids are essential for vision, embryonic development, the immune response, cellular growth, differentiation, and cellular death [101]. Retinoic acid, one of the active forms of vitamin A, stimulates the secretion of growth hormone, which has downstream effects on IGF1 levels, as discussed above. Moreover, vitamin A deficiency (VAD) is a common issue in T21 patients. A study by Ferraz et al. [102] explored the relationship between IGF1 levels and VAD in children with T21 (ages 24–72 months). While the study found no direct association between VAD and IGF1 deficiency, a positive correlation between serum retinol levels and IGF1 was observed, suggesting that impaired retinoid metabolism might contribute to the disruptions in IGF1 signaling observed in T21. Retinoids also influence adult neuronal functioning, including memory and plasticity with reduced levels of retinoic acid being reported in AD patients, based on indirect evidence [103]. Given the high prevalence of AD in individuals with T21, further research into the role of retinoid transport and metabolism in T21 and its potential connection to AD is warranted.

The enrichment analysis identified several modifications in disease-associated pathways, as defined by the Reactome database, further confirming previous observations of altered pathways in T21. Notably, pathway modifications related to glycosaminoglycan (GAG) metabolism were observed specifically in the AF of T21 samples. DEPs with elevated expression in T21 AF samples were involved in heparan sulfate (HS) metabolism, chondroitin sulfate (CS) or dermatan sulfate (DS) metabolism, and DS biosynthesis (Table S4, Figure S2). HS, a glycosaminoglycan forming proteoglycans, plays a crucial role in biological processes, including angiogenesis, blood coagulation, and tumor metastasis [71]. Perlecan, also known as heparan sulfate proteoglycan 2 (HSPG2), is a component of the ECM and has been reported at elevated levels in the AF of T21 pregnancies [29]. Perlecan binds and cross-links ECM components and is known to play a role in chondrogenesis, bone development, and cartilage integrity, though its physiological role in AF remains unclear [59]. Additionally, CS and DS are commonly associated with atherosclerosis, nerve development and repair, inflammation, tumor growth, and metastasis [80]. Modifications of the enzymes involved in the biosynthesis of glycosaminoglycans are important in Ehlers–Danlos syndrome, joint dislocations, short stature, spondyloepiphyseal dysplasia with congenital joint dislocations, spondyloepimetaphyseal dysplasia with joint laxity type 1, congenital heart defects, and Temtamy preaxial brachydactyly syndrome. While congenital heart defects and joint laxity are common in T21 patients, the co-occurrence of T21 and Ehlers–Danlos syndrome is rare, and no established correlation exists between the two conditions [104].

Pathways associated with diseases of hemostasis were predominantly observed in maternal plasma, along with pathways related to signal transduction mediated by growth factors and second messengers—specifically, oncogenic MAPK signaling. MAPKs are protein kinases that control intracellular processes, such as gene expression, metabolism, proliferation, differentiation, and apoptosis, as part of normal physiology, being mainly studied in the context of oncogenesis, tumor progression, and drug resistance [105]. MAPK pathways in T21 patients have been primarily studied to enhance antitumor treatment efficacy in patients with B cell acute lymphoblastic leukemia [106] or to assess MAPK activity in the brains of T21 and Alzheimer’s disease patients [107].

Table 2 summarizes the key molecular pathways implicated in Down syndrome (T21), emphasizing their normal biological functions and the observed or potential alterations in T21. While direct evidence for some pathways remains limited, numerous pathways—particularly those involved in signaling, immune functions, extracellular matrix organization, and metabolic processes—show promising associations with the clinical features of T21.

### 4.4. Pathway Associations of Literature-Reported Differentially Expressed Metabolites

Regarding the metabolomic pathways of significant differentially expressed metabolites (DEMs) in T21, brief discussions on this topic are included in the description of each metabolomic study outlined in the previous section. Additionally, a comprehensive list of DEMs reported in the literature is included in Table S3 along with HMDB [108] extracted data describing the metabolite function and roles.

For AF samples, the common findings across studies suggest that pathways involved in amino acid metabolism (glutamate, alanine, aspartate, histidine, arginine, proline), lipid metabolism (phospholipids, fatty acids), carbohydrate metabolism (fructose, mannose, galactose, pentose, glycogen, disaccharides, and oligosaccharides), and neural and muscular development seem to be affected in T21 cases [49,50,51,52].

The possibly pathways affected by the DEMs in the maternal serum and plasma of T21 pregnancies include pathways involved in lipids synthesis (with important roles in myelinization and brain development), muscular and neurological disorders (hypotonia, learning disabilities, seizure disorders, brain atrophy), neurological development (oxidative stress), growth delay, and DNA methylation [53,54,55,56,57]. 3-Hydroxybutyrate and 2-hydroxybutyrate were found to have increased levels in plasma and serum samples of T21 pregnancies in many studies [53,55,56,57]. 3-Hydroxybutyrate represents a source of energy for the brain in cases of hypoglycemia, while 2-hydroxybutyrate is increased in oxidative stress, with both metabolites being involved in brain development [53,55]. 3-Hydroxyisovalerate represents another metabolite with different expression in T21 cases showing inconsistent expression levels among different studies, either elevated [53,56,57] or reduced [55]. Generally, it is a biomarker of biotinidase deficiency associated with muscular and neurological disorders [53].

Fewer metabolomic studies were conducted using maternal urine as the sample. Lower levels of progesterone were found, suggesting a lower activity of progesterone from the feto-placental unit, and higher levels of dihydrouracil, associated with disfunction or deficiency of dihydropyrimidinase, manifesting as grow retardation and facial dysmorphism [58].

## 5. Conclusions

This review has explored the advancements in mass spectrometry (MS)-based omics technologies for identifying biomarkers that could enhance non-invasive prenatal screening for Down syndrome. The advantages of MS in analyzing complex biological samples were discussed, which makes this technique an ideal platform for biomarker discovery. By reviewing MS-based studies that analyze maternal samples from T21 pregnancies, we identified key proteins and metabolites with potential as biomarkers for T21, while also providing an overview of the methodologies employed. The findings emphasize that MS analytical techniques have proven to be suitable for detecting novel non-invasive biomarkers for T21, offering new insights into the complex pathophysiology of the disorder and holding the potential to improve the detection rates of this fetal aneuploidy.

The literature-reported differentially expressed proteins were extracted and analyzed, providing valuable insights into the molecular mechanisms altered in T21 revealing disruptions in pathways related to signal transduction, ECM organization, protein metabolism, innate immune system functions, and retinoid metabolism. While significant progress has been made in understanding the molecular underpinnings of T21, there are gaps in elucidating the direct involvement of several biological pathways in the pathophysiology of T21. Given that T21 is associated with chronic low-grade inflammation, exploring the NLRP3 inflammasome pathway could provide new insights into the underlying immune mechanisms and potentially identify novel biomarkers for T21. Similarly, disruptions in integrin–MAPK signaling in T21 pregnancies may indeed be a key factor contributing to placental insufficiency. Additionally, the identification of DEPs involved in scavenger-receptor-mediated ligand uptake across various biological matrices offers a potential area of interest for further research.

However, several challenges remain, particularly with validating the identified biomarkers in larger cohorts and standardizing data across various MS methodologies. To make these findings clinically applicable, continued research is essential to refine and validate these biomarkers

Moreover, MS-based omics studies, particularly those focusing on maternal urine samples, hold significant potential. These non-invasive samples, though underexplored so far, could provide an alternative approach for biomarkers of T21. Future studies should prioritize validating the identified proteins and metabolites for clinical use and investigate the potential integration of these omics-based techniques into standard, non-invasive prenatal screening protocols for T21. By doing so, we could pave the way for a more accurate, accessible, and timely diagnoses, thereby enhancing prenatal care and the management of T21 and related conditions.

## Figures and Tables

**Table 1:** Overview of literature MS-based studies applied in prenatal screening for Down Syndrome.

Biological Matrix Studied	Study Design	Methods Implemented	Main Results (in T21)	Ref.
Proteomics studies				
Amniotic fluid cells (amniocytes)	T21 = 3; Control = 4 [16–18 gestational weeks]	In vitro culturing of amnion cells 2-DE + MALDI-MS analysis	99 proteins identified; aberrant expression linked to purine, carbohydrate, intermediary, and amino acid metabolism in amnion cells from T21 patients.	[26]
	T21 = 3; Control = 5 [15–21 gestational weeks]	SCX fractionation via 2D-LC-MS/MS RP-nanoLC-Orbitrap-MS Protein quantification via RP-nanoLC-TQ-MS (SRM)	Over 4900 proteins identified from primary amniocytes (proteomic discovery), with at least 900 dysregulated in T21 (quantitative analysis). Dysregulated proteins in T21 were linked to cell morphology, hematological development, immune response, lipid metabolism, cardiovascular disease, genetic and metabolic disorders, protein degradation, embryonic development, cancer, neurological diseases, and tissue development.	[27]
	T21 = 18; Control = 20 [18–22 gestational weeks]	2-DE + MALDI-TOF-MS Western Blot (WB) analysis	Six proteins were significantly upregulated in T21 amniocytes: calumenin, nucleophosmin, elongation factor 1-beta, cathepsin D, platelet-activating factor acetylhydrolase IB subunit beta, and 14-3-3 protein beta/alpha. Western Blot (WB) analysis confirmed alterations in nucleophosmin and cathepsin D.	[28]
Amniotic fluid supernatants (AFS)	T21 = 6; Control = 12 [17 gestational weeks]	2-DE + MALDI-MS analysis Nano-ESI-MS/MS Western blot (WB)	Seven proteins were differentially expressed in pregnancies with T21 fetuses. Five of these proteins were upregulated in T21 cases, SFRS-4 was detected only in T21, and a 40% decrease in IBP-1 concentration was observed in amniotic fluid (AFS) from T21 cases.	[29]
	T21 = 19; T18 = 17; Control = 34 [15–20 gestational weeks]	2D chromatography separation and fraction selection In-gel and in-solution digestion + MALDI-TOF-MS analysis Western blot (WB) analysis	Proteins with significant differential expressions in T21 included apolipoprotein A1, antitrypsin, prealbumin (transthyretin), and transferrin. Apolipoprotein A1 levels were significantly decreased in amniotic fluid (AFS) of both T18 and T21, while antitrypsin, transferrin, and prealbumin levels were increased in T21 AFS. Functional network analysis linked dysfunction of cholesterol metabolism to T21.	[30]
	T21 = 4; Control = 6 [15–18 gestational weeks]	Affinity chromatography to remove albumin and immunoglobulin G In-gel digestion + LC-ESI-MS/MS analysis Western blot (WB) analysis	Forty-four AFS proteins were differentially expressed between T21 and normal cases, with six unique to T21. Western blot (WB) analysis confirmed apolipoprotein A-II (apo-II) and alpha-fetoprotein (AFP) as potential diagnostic tools for T21.	[31]
	T21 = 10; Control = 15 [16–20 gestational weeks]	Immunoglobulin depletion + 2D-LC fractionation followed by MS/MS (LTQ-Orbitrap MS) ELISA for candidate biomarkers on maternal serum samples	Sixty proteins showed greater than 2-fold changes in T21. Top pathways for decreased proteins in T21 were associated with organ morphology and reproductive system development and function. Amyloid precursor protein and tenascin-C were evaluated via ELISA in serum samples, showing increased levels in T21 cases.	[32]
	T21 = 9 Control = 9 [15–17 gestational weeks]	2-DE Western blot (WB) analysis LC-MS/MS for protein identification	Proteins involved in iron homeostasis (ceruloplasmin and transferrin), lipid metabolism (zinc-alpha-2-glycoprotein, retinol-binding protein 4, and apolipoprotein A1), and inflammation (complement C9, α-1B-glycoprotein, collagen α-1V chain) were identified as critically relevant to the clinical outcome of T21.	[33]
	T21 = 10; Control = 10 [16–20 gestational weeks]	SRM assay developed to test thirteen previously identified candidate proteins in amniotic fluid ELISA for candidate biomarkers	Bile-salt-activated lipase, mucin-13, carboxypeptidase A1, and dipeptidyl peptidase 4 showed decreased levels in amniotic fluid of T21 cases, while matrix metalloproteinase-2 levels were significantly increased. In serum samples, matrix metalloproteinase-2 levels showed no significant difference between control and T21 groups.	[34]
	T21 = 17; Control = 37 [15–22 gestational weeks]	LC-SRM-MS	Five proteins (bile salt-activated lipase, carboxypeptidase A1, mucin-13, chloride channel accessory 1, and mucin-5AC) were significantly downregulated in T21 cases, while one protein (hyaluronan and proteoglycan link protein 1) was upregulated.	[35]
Maternal serum	T21 = 14; Control = 15 [8–13 gestational weeks]	Bead-based multiplexed immunoassays	Seven potential biomarkers were selected for further analysis: alpha fetoprotein, epidermal growth factor, extracellular rage binding protein, eotaxin, haptoglobin, insulin, and lipoprotein A. None of these biomarkers were fully discriminatory between T21 cases and controls.	[36]
	T21 = 27; Control = 27 [12 gestational weeks]	Bead-based multiplexed immunoassays	Prediction values were obtained for current screening markers (pregnancy-associated plasma protein A, free beta human chorionic gonadotrophin, and nuchal translucency) and seven previously identified markers based on concentration ratios between T21 and controls. Validation of these biomarkers confirmed epidermal growth factor for further consideration as a T21 screening marker.	[37]
	T21 = 24; Control = 21 [first trimester pregnancies]	LC-MS/MS; multiplexed SRM assay	Over 300 proteins were identified, with 12 selected for further development into multiplexed SRM assays. IPA analysis revealed that differentially expressed proteins are implicated in humoral immune response, cardiovascular system development, cellular growth and proliferation, and lipid metabolism.	[38]
	T21 = 50; Control = 25 [11–13 gestational week]	ELISA	In pregnancies with fetal T21, maternal age, fetal nuchal translucency thickness, and serum free beta human chorionic gonadotrophin were increased, while serum pregnancy-associated plasma protein A was decreased. No significant differences were found between T21 cases and controls in any of the biomarkers.	[39]
	T21 = 6; Control = 6 [16–19 gestational weeks]	2-DE and MALDI-MS; ELISA for candidate biomarkers	Twenty-nine proteins were identified in maternal serum from pregnancies with T21-affected fetuses. These proteins were involved in biological regulation, metabolic processes, cellular processes, and response to stimulus. Ceruloplasmin and complement factor B expression were confirmed using ELISA.	[40]
Maternal plasma	T21 = 8; Control = 12 [16–18 gestational weeks]	2-DE + MALDI-TOF-MS Western blot (WB) confirmation	Nine DEPs were identified in maternal plasma of women with T21 fetuses, associated with fetal growth and development: transthyretin, ceruloplasmin, afamin, alpha-1-microglobulin, apolipoprotein E, serum amyloid P-component, histidine-rich glycoprotein, and alpha-1-antitrypsin (upregulated), and clusterin (downregulated). Apolipoprotein E and serum amyloid P-component levels were confirmed via WB analysis.	[41]
	T21 = 6; Control = 6 [11–14 gestational weeks]	Immunodepletion of high-abundance plasma proteins SCX fractionation + Nano-LC MALDI-MS analysis	A total of 178 proteins were quantified. Twenty-eight proteins were upregulated in T21, linked to signaling and immunity, while 22 were downregulated, related to cell adhesion and the extracellular matrix. Panther analysis showed 13.3% of proteins in T21 samples are involved in Alzheimer’s disease pathways, and over 40% are linked to integrin signaling.	[42]
	T21 = 14; Control = 14 [10–14 gestational weeks]	2D DIGE + MALDI-TOF-MS ESI Q-TOF MS/MS Western blot (WB) analysis	No DEPs were observed in the first trimester. In the second trimester, increased levels of ceruloplasmin, inter-alpha-trypsin inhibitor heavy chain H4, complement proteins (C1s subcomponent, C4-A, C5, C9), and kininogen 1 were detected in T21 maternal plasma. Ceruloplasmin expression in maternal plasma was confirmed via WB.	[43]
	T21 = 19; Control = 19 [10–20 gestational weeks]	SRM assay development for the quantification of two biomarkers	Significant differences in maternal plasma levels of serum amyloid-P and C1-inhibitor were observed between T21-affected and high-risk normal pregnancies in both the first and second trimesters.	[44]
	T21 = 28; Control = 53 [10–20 gestational weeks]	SELDI-TOF analysis ProteinChip^®^ Q spin columns SDS-PAGE nano-LC-QTOF-MS analysis Western blot (WB) analysis	Plasma C1-inhibitor was significantly elevated in T21 vs. control (10–14 weeks) via SELDI-TOF MS analysis. Transthyretin, serum amyloid P, and complement C3 showed statistically significant changes in T21 vs. control (14–20 weeks).	[45]
Umbilical cord blood	T21 = 6; Control = 11	iTRAQ, SCX fractionation + MALDI TOF/TOF	A total of 505 proteins were identified, with 13 upregulated and 6 downregulated in T21. Apolipoprotein E, complement factor B, amyloid P component, matrin-3, and osteopontin were found to be relevant to T21, with the first three notably upregulated. The panel was proposed as potential T21 biomarkers.	[46]
Placenta	T21 = 19; Control = 17 [18–24 gestational weeks]	2D-DIGE + MALDI TOF/TOF MS analysis	Annexin A2, endoplasmic reticulum protein, copper-zinc superoxide dismutase, proteasome subunit alpha type-2, heat shock protein beta-1, peptidyl-prolyl cis-trans isomerase, and fibrinogen beta chain were upregulated in T21 placenta. Copper-zinc superoxide dismutase, endoplasmic reticulum protein, and heat shock protein beta-1 were linked to reactive oxygen species damage resistance and neurogenesis. Peroxiredoxin-6, enoyl-CoA hydratase, and protein disulfide isomerase A3 were downregulated.	[47]
Metabolomics studies				
Amniotic fluid supernatants (AFS)	T21 = 22; Control = 41 [15–17 gestational weeks]	GC-MS analysis	28 organic acid metabolites were quantified via GC-MS in T21 vs. control samples. Increased markers of riboflavin deficiency (5-hydroxycaproate, methylsuccinate, α-ketoglutarate, adipate) were found in T21 AFS. Elevated phenylpyruvate levels in T21 indicated involvement in neurotransmitter metabolism.	[48]
	discovery set: T21 = 10; Control = 10; validation set: T21 = 15; Control = 15; [17 gestational weeks]	LC-HRMS (Q-TOF) in four conditions (HILIC and RP, positive and negative) Metabolites validated using standards	Notable alterations in the metabolites of coproporphyrin III, pregnenolone sulfate, taurochenodeoxycholate, L-arginine, taurocholate, hydrocortisone, L-histidine, glycocholic acid, L-glutamate, and L-glutamine. The primary pathway modifications in T21 fetuses were related to amino acid metabolism, bile secretion, neuroactive ligand-receptor interactions, and galactose metabolism.	[49]
	T21 = 21; Control = 21; [18 gestational weeks]	UPLC-MS/MS (Orbitrap) in four conditions (HILIC and RP, positive and negative)	Key metabolites associated with four primary metabolic pathways were identified as significant in differentiating T21: gamma-glutamyl amino acids, steroid hormone derivatives, polyamines (notably N1, N12-diacetylspermine), and glycerol derivatives from phospholipid breakdown. In T21 cases, steroid hormone and gamma-glutamyl amino acid levels were generally decreased, whereas N1, N12-diacetylspermine, and phospholipid derivatives were elevated.	[50]
	T21 = 20; Control = 20; [17–24 gestational weeks]	2D LC-MS/MS analysis	Significant alterations were observed in metabolites, particularly lipid molecules, organic acids, and nucleotides. These changes were associated with pathways related to energy metabolism, amino acid metabolism, organic acid metabolism, and steroid hormone synthesis.	[51]
	T21 = 13; Control = 13; [15–17 gestational weeks]	UHPLC-MS (Q-TOF)—RP-C18—in positive and negative modes	An increase in diacetylspermine levels was observed in T21, alongside a significant decrease in p-cresol sulfate, methylhistidine, and hexanoylcarnitine.	[52]
Maternal serum	T21 = 30; Control = 60; [11–13 gestational weeks]	NMR spectroscopy	VIP analysis identified 3-hydroxybutyrate, 3-hydroxyisovalerate, and 2-hydroxybutyrate as the most effective metabolites for distinguishing T21 cases from controls. These metabolites are linked to oxidative stress, impaired myelination, and neurotoxicity in T21 individuals.	[53]
Maternal plasma	T21 = 12; Control = 15; [15–18 gestational weeks]	UHPLC-Q-TOF-MS, with ESI	The concentrations of five fatty acid amide metabolites were significantly reduced in the plasma of pregnancies with T21. Most of these metabolites were linked to fetal brain and central nervous system development. The study suggests these metabolites as potential new markers for non-invasive prenatal diagnosis of fetal T21.	[54]
	T21 = 21; Control = 32 [17–19.4 gestational weeks]	GC-MS and LC-Q-TOF-MS	Two complementary MS-driven techniques were used to profile the metabolites in T21 and normal maternal plasma samples. Elevated levels of 3-hydroxybutyric acid and 2-ketoisocaproic acid were observed in the T21 group, while beta-alanine, threonic acid, oxalic acid, alpha-tocopherol, uracil, 2-piperidone, and creatinine showed reduced levels. The study also revealed a decrease in lipid-related metabolites in women carrying T21 fetuses.	[55]
	T21 = 21; Control = 32 [11–15 gestational weeks]	GC-MS	Metabolites, such as 2-hydroxybutyric acid, 3-hydroxybutyric acid, β-hydroxyisovaleric acid, uracil, glutamic acid, maltose, and melezitose, were identified as potential biomarkers for prenatal T21 screening.	[56]
	T21 = 17; Control = 30 [gestational age not mentioned]	LC-MS/MS	An LC-MS/MS technique was developed to analyze two potential biomarkers: 3-hydroxybutyric acid and 3-hydroxyisovaleric acid. Increased levels of both biomarkers were found in T21 pregnancies.	[57]
Maternal urine	T21 = 23; non-T21 aneuploidies= 6; Control = 93; [11–17 gestational weeks]	ZIC-HILIC and RPLC coupled to hybrid ion trap- TOF MS	Two stationary phases were tested for urinary metabolome coverage, with ZIC-HILIC-MS outperforming RPLC-MS. Significant alterations in maternal urinary concentrations of progesterone and dihydrouracil were linked to the presence of a T21 fetus. A screening test based on metabolomics profiling successfully identified approximately 87% of T21 pregnancies between 9 and 23 weeks of gestation using HILIC-MS, with no false positives reported.	[58]
	T21 = 20; Control = 20; [17–24 gestational weeks]	2D LC-MS/MS analyses	The study aimed to explore metabolomic changes in the amniotic fluid and urine of pregnant women carrying fetuses with T21. The analysis revealed a significantly lower number of differential metabolites in urine compared to amniotic fluid, with phenylalanine and glycerophospholipid metabolism pathways being enriched among the urine metabolites.	[51]

Abbreviations: MALDI, matrix-assisted laser desorption ionization; SCX, strong cation exchange; 2D-LC, two-dimensional liquid chromatography; TQ-MS, triple quadrupole mass spectrometry; SRM, selected reaction monitoring; TOF-MS, time-of-flight mass spectrometry; WB, Western blot; ELISA, enzyme-linked immunosorbent assay; IPA, ingenuity pathway analysis; 2D DIGE, two-dimensional difference gel electrophoresis; SELDI, surface-enhanced laser desorption/ionization; SDS-PAGE, sodium dodecyl-sulfate polyacrylamide gel electrophoresis; iTRAQ, multiplexed isobaric tagging technology for relative quantitation; NMR, nuclear magnetic resonance; ZIC, zwitterionic hydrophilic interaction.

**Table 2:** Summary of key molecular pathways and their implications in Down syndrome.

Pathway Group	Pathway/Process	Implications in T21
Signal transduction pathways	Signaling by platelet-derived growth factor (PDGF)	Direct evidence: no Normal function: stimulates growth and motility of connective tissue cells, neurons, and capillary endothelial cells. Key findings related to T21: linked to transient abnormal myelopoiesis in T21 newborns, which can progress to hepatic necrosis and failure. Elevated PDGF expression observed in megakaryoblasts of T21 patients with hepatic disease.
	Signaling by MET	[Through integrins interactions e.g., PTK2] Direct evidence: no Normal function: plays a key role in promoting cell motility and migration, particularly in neuronal and connective tissue processes. Key findings related to T21: altered PTK2 expression in T21 has been documented, particularly in nervous system and connective tissue processes.
	Integrin signaling	Direct Evidence: yes Normal function: regulates key cellular processes, such as proliferation, differentiation, and migration, essential for embryonic and placental development. Key findings related to T21: disruptions in integrin–MAPK signaling may contribute to placental insufficiency and associated complications.
	Neural cell adhesion molecule (NCAM) signaling for neurite out-growth	Direct evidence: no Normal function: essential for nervous system development and synaptic plasticity. Key findings related to T21: disruptions in NCAM signaling pathways may be linked to neurodevelopmental delays in T21.
	Regulation of NR1H2 and NR1H3	Direct evidence: no Normal function: regulates cholesterol efflux, lipid metabolism, and anti-inflammatory responses. Protects against atherosclerosis and neurodegeneration. Key findings related to T21: altered cholesterol transport and efflux in T21 may contribute to cardiovascular dysfunction and neurodegeneration.
Hemostasis	Platelet activation, signaling, and clotting cascade	Direct evidence: yes Normal function: involved in blood coagulation, wound healing, and maintaining hemostasis. Key findings related to T21: elevated thrombosis risk in T21 patients, suggesting a prothrombotic state.
Extracellular matrix (ECM) organization	Collagen formation, collagen degradation, ECM proteoglycans	Direct evidence: yes Normal function: ECM is crucial for structural support, tissue integrity, and signaling processes. Key findings related to T21: disturbances in ECM contribute to congenital anomalies, like heart defects and hypotonia. Overexpression of collagen VI has been observed in T21 fetal hearts.
Protein metabolism	Amyloid formation	Direct evidence: yes Normal function: amyloid proteins are involved in protein folding and function, but accumulation leads to neurodegenerative diseases. Key findings related to T21: increased risk of Alzheimer’s disease (AD) in T21, with evidence of fibrillar β-amyloid peptide deposits.
	Post-translational protein modifications	[Through phosphorylation pathways] Direct evidence: no Normal function: phosphorylation regulates protein function, including activity, localization, and interactions. Key findings related to T21: disruptions in phosphorylation-related pathways may contribute to protein dysfunction in T21
	Regulation of insulin-like growth factor (IGF) transport and uptake by insulin-like growth factor binding proteins (IGFBPs)	Direct evidence: no Normal function: IGF signaling regulates growth, development, and apoptosis. Key findings related to T21: reduced IGF1R expression may impair cardiomyocyte proliferation, contributing to congenital heart defects and short stature in T21. Lower IGF1 signaling is linked to inflammation, neurodegeneration, and short stature. Correlations have been found between neurodegeneration biomarkers (e.g., NfL, UCHL1, GFAP) and IGF signaling disruptions.
Innate immune system	Complement cascade pathways	Direct evidence: yes Normal function: the complement system is crucial for immune responses, inflammation, and pathogen clearance. Key findings related to T21: altered complement protein expression in T21 may be relevant to early-onset AD and is associated with chronic infections, inflammation, accelerated aging, obesity, and cognitive decline.
	NLRP3 inflammasome	Direct evidence: no Normal function: NLRP3 activation promotes the release of pro-inflammatory cytokines (IL-1β, IL-18), contributing to immune responses. Key findings related to T21: NLRP3 activation promotes pro-inflammatory cytokine release (IL-1β, IL-18), contributing to inflammatory and autoimmune diseases. T21 patients exhibit an inflammatory profile with elevated IL-1β levels.
Transport of small molecules	Plasma lipoprotein metabolism [APOA1 and APOE expression]	Direct evidence: no Normal function: APOA1 is involved in lipid metabolism and cholesterol efflux, while APOE influences lipid transport and brain function. Key findings related to T21: APOA1 is linked to amyloidosis. Higher APOE expression observed in T21 maternal plasma. The presence of the ApoE-ε4 allele in T21 pregnancies is linked to increased blood cholesterol levels, which may impair ovarian follicle microcirculation and fertility.
Vesicle-mediated transport	Ligand binding and uptake by scavenger receptors	Direct evidence: no Normal function: scavenger receptors mediate the clearance of oxidized LDL and pathogens, contributing to immune defense and cellular homeostasis. They are expressed in multiple tissues, including the placenta and heart. Key findings related to T21: altered scavenger receptor expression in T21 may impact immune function and oxidative stress management.
Retinoid metabolism	Retinoid transport and signaling	Direct evidence: no Normal function: retinoids play a role in vision, embryonic development, immune function, and cellular growth. Retinoic acid stimulates growth hormone secretion, affecting IGF1 levels. Retinoids influence neuronal function, memory, and plasticity. Key findings related to T21: vitamin A deficiency (VAD) is common in T21. Reduced retinoic acid levels have been reported in AD, which is highly prevalent in T21.
Disease-associated pathways	Disease of glycosaminoglycan (GAG) metabolism	Direct evidence: no Normal function: GAGs are essential for angiogenesis, coagulation, and ECM integrity. Key findings related to T21: modifications in GAG biosynthesis enzymes are linked to conditions, such as Ehlers–Danlos syndrome, congenital heart defects, and skeletal abnormalities observed in T21.
	Diseases of hemostasis	Direct evidence: no Normal function: involves the regulation of blood coagulation and prevention of excessive bleeding. Key findings related to T21: T21 is associated with altered clotting pathways, contributing to an increased thrombotic risk.
	Oncogenic MAPK signaling	Direct evidence: no Normal function: MAPK signaling is essential for cell growth, differentiation, and survival. Key findings related to T21: studied in T21 patients for its role in leukemia and neurodevelopment. MAPK activity is linked to acute lymphoblastic leukemia in T21.

Notes: Direct evidence: “Yes” indicates that the finding is directly supported by studies on T21; “No” suggests indirect evidence or extrapolation from related studies.

## Supplementary Materials

The following supporting information can be downloaded at: https://www.mdpi.com/article/10.3390/life15050695/s1, Figure S1: Schematic representation of literature review methodology implemented within this manuscript; Figure S2: Enrichment analysis of differentially expressed proteins showing higher abundance in T21 reported for three different matrices; Figure S3: Enrichment analysis of differentially expressed proteins showing lower abundance in T21 reported for three different matrices; Table S1: Articles included in the review process; Table S2: Combined list of proteins reported in the literature as differentially expressed in Down syndrome; Table S3: Combined list of metabolites reported in the literature as differentially expressed in Down syndrome; Table S4: Enrichment analysis conducted with the ClueGo and Cluepedia Cytoscpae apps using the Reactome database.
