# Supplementary material for: Non-Invasive Prenatal Screening for Down Syndrome: A Review of Mass-Spectrometry-Based Approaches
Source: Life (Basel). 2025 Apr 24;15(5):695. doi: 10.3390/life15050695 (PMC12112985; doi:10.3390/life15050695)
Supplement: Supplementary file 1 [file life-15-00695-s001.zip › Supplementary figures.pdf]

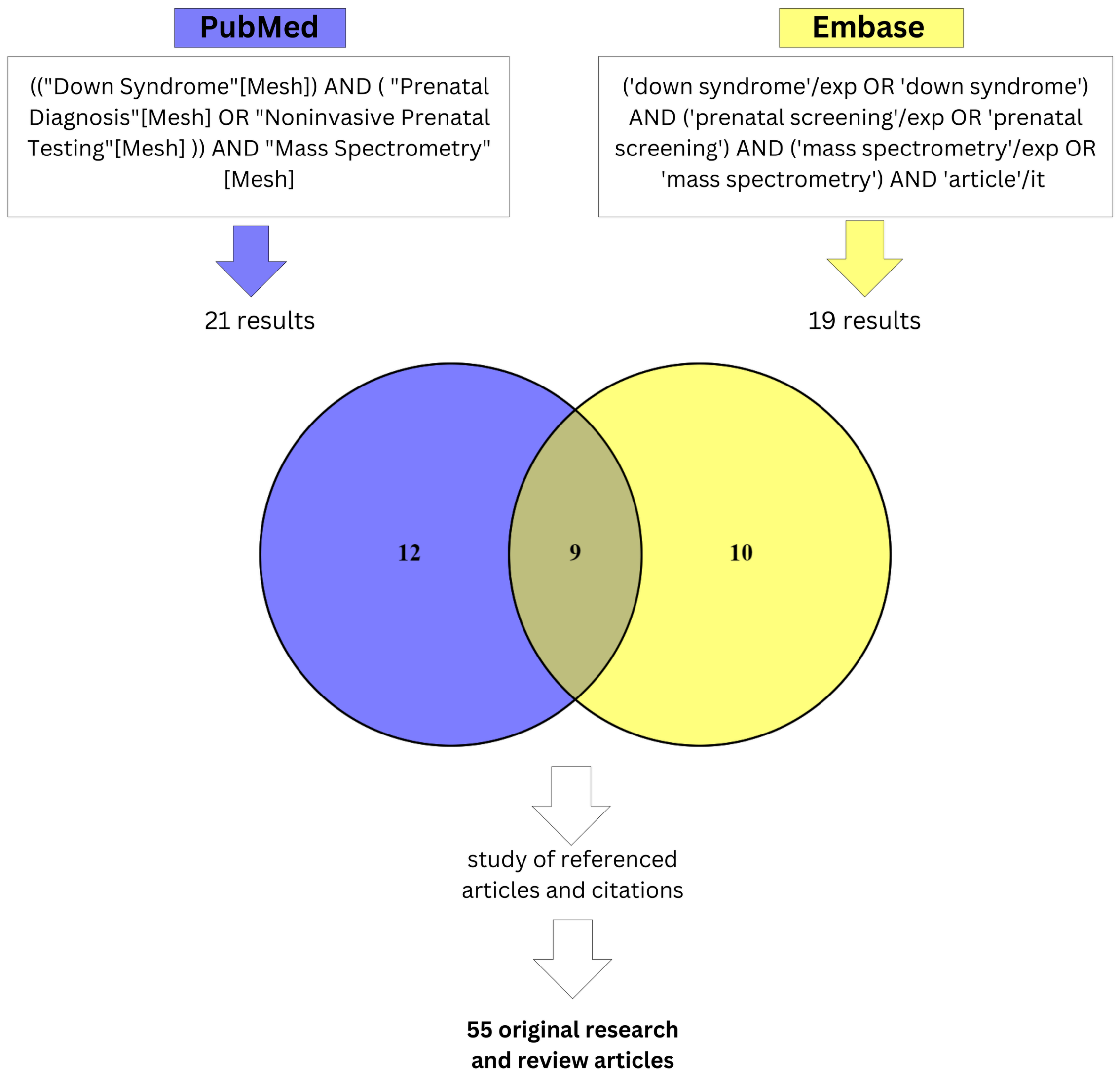

Figure S1: Schematic representations of literature review methodology implemented within this manuscript.

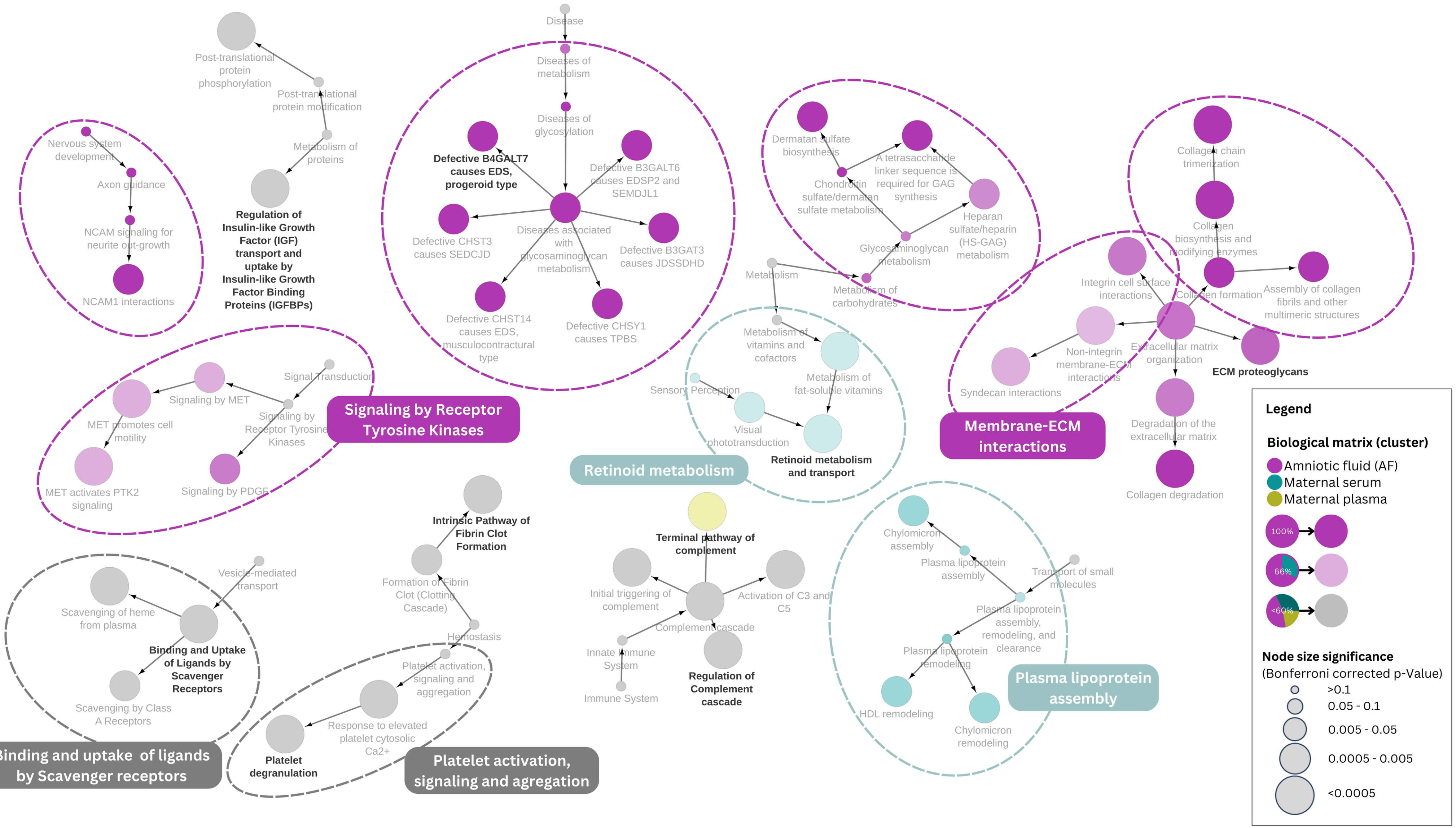

Figure S2: Enrichment analysis of differentially expressed proteins showing higher abundance in T21 reported for three different matrices. Network vizualization and enrichment analysis were obtained using ClueGO and Cluepedia apps for Cytoscape . Bonferroni corrected p-value cutoff: 0.005, min 3 genes/term; the node size shows the term significance: the biggest terms are the most significant ones. The node color shows the proportion of genes from each cluster that are associated with the term. Specific terms: >60% genes from a cluster, node color is based on the proportion of genes and is a gradient white of the cluster color; Common terms: <60%, node color: gray. The hierarchical relationship between pathways was visually represented with arrows. Note on clustering: The clustering in the figure groups pathways according to their parent nodes. While the local distance between clusters is visually presented, it does not represent a specific biological or computational relationship but serves to highlight the pathway groupings based on their parent nodes.

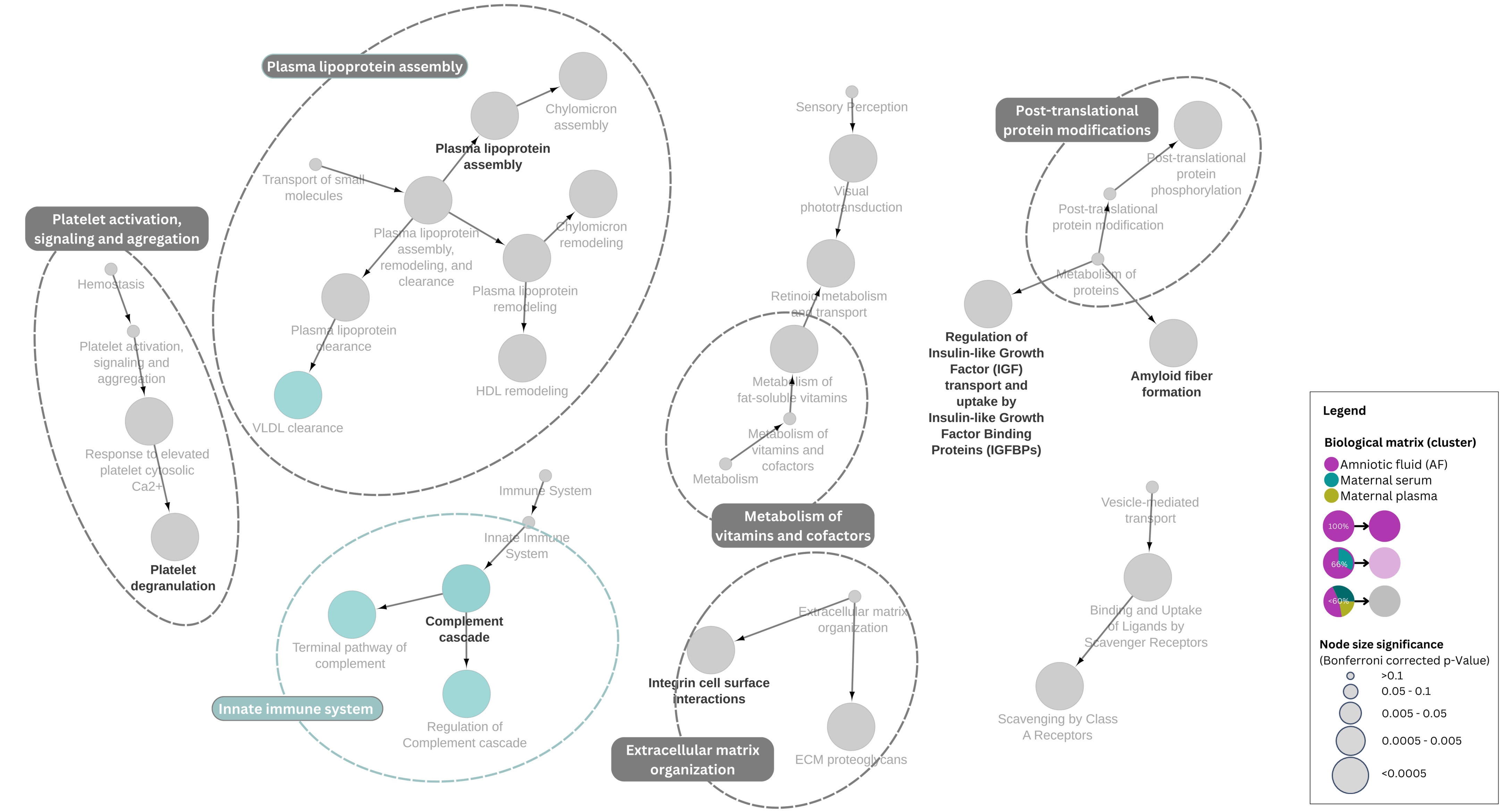

Figure S3: Enrichment analysis of differentially expressed proteins showing lower abundance in T21 reported for three different matrices. Network vizualization and enrichment analysis were obtained using ClueGO and Cluepedia apps for Cytoscape. Bonferroni corrected p-value cutoff: 0.005, min 3 genes/term; the node size shows the term significance: the biggest terms are the most significant ones. The node color shows the proportion of genes from each cluster that are associated with the term. Specific terms: >60% genes from a cluster, node color is based on the proportion of genes and is a gradient white of the cluster color; Common terms: <60%, node color: gray. The hierarchical relationship between pathways was visually represented with arrows. Note on clustering: The clustering in the figure groups pathways according to their parent nodes. While the local distance between clusters is visually presented, it does not represent a specific biological or computational relationship but serves to highlight the pathway groupings based on their parent nodes.
